# Soil-transmitted helminth infections among pre-school aged children in Gamo Gofa zone, Southern Ethiopia: Prevalence, intensity and intervention status

**DOI:** 10.1371/journal.pone.0243946

**Published:** 2020-12-15

**Authors:** Mekuria Asnakew Asfaw, Tigist Gezmu, Teklu Wegayehu, Alemayehu Bekele, Zeleke Hailemariam, Nebiyu Masresha, Teshome Gebre

**Affiliations:** 1 Collaborative Research and Training Centre for NTDs, Arba Minch University, Arba Minch, Ethiopia; 2 Department of Biology, College of Natural Sciences, Arba Minch University, Arba Minch, Ethiopia; 3 School of Public Health, College of Medicine and Health Sciences, Arba Minch University, Arba Minch, Ethiopia; 4 Ethiopian Public Health Institute, Addis Ababa, Ethiopia; 5 The Task Force for Global Health, International Trachoma Initiative, Addis Ababa, Ethiopia; Dokkyo Medical University, JAPAN

## Abstract

**Background:**

Soil-transmitted helminths (STH), i.e., *Ascaris lumbricoides*, *Trichuris trichiura* and hookworms are among the most prevalent Neglected Tropical Diseases (NTDs) in Ethiopia. Although pre-school aged children pay a high morbidity toll associated with STH infections, evidence on prevalence, intensity and intervention status is lacking in Ethiopia. This study, therefore, aimed to address these gaps to inform decision made on STH.

**Methods:**

We did a community-based cross-sectional study in five districts of Gamo Gofa zone, Southern Ethiopia; in January 2019. Data were collected using pre-tested questionnaire, and the Kato-Katz technique was used to diagnose parasites eggs in stool. Then, collected data were edited and entered into EpiData 4.4.2, and exported to SPSS software (IBM, version 25) for analysis.

**Results:**

A total of 2462 PSAC participated in this study. Overall, the prevalence of STH was 23.5% (578/2462) (95% confidence interval (CI) = 21.8%–25.2%). As *caris lumbricoides* was the most prevalent (18.6%), followed by *Trichuris trichiura* (9.2%), and hookworms (3.1%). Of the total, 7.4% PSAC were infected with two STH species. Most of the positive cases with STH showed low infection intensities, while 15.1% *ascariasis* cases showed moderate infection intensities. The study found that 68.7% of PSAC were treated with albendazole. Also, household’s level data showed that 39.4% used water from hand-dug well; 52.5% need to travel ≥30 minutes to collect water; 77.5% did not treat water, and 48.9% had no hand washing facility. In addition, almost 93% care givers achieved less than the mean knowledge and practice score (≤5) on STH prevention.

**Conclusions:**

This study showed that significant proportions of pre-school aged children are suffering from STH infections despite preventive chemotherapy exist at the study area. Also, gaps in the interventions against STH were highlighted. Thus, a call for action is demanding to eliminate STH among PSAC in Ethiopia by 2030.

## Introduction

Soil-transmitted helminth (STH), i.e., *Ascaris lumbricoides*, *Trichuris trichiura* and hookworms infections are among the most common Neglected Tropical Diseases (NTDs) [1]. It is prevalent mainly in tropical and subtropical areas where water supply, hygiene and sanitation infrastructures are inadequate [2–4]. Moderate and heavy infection intensities of STH are associated with chronic harmful effects on vitamin A and iron status, physical, intellectual, and cognitive development in pre-school aged children (PSAC); these morbidities not only take a huge toll on the health of children, but have also been shown to affect economic development of a nation [5, 6].

Globally, over 2 billion people are affected with STH, where ascariasis accounts for almost 1.2 billion infections, while trichiurasis, and hookworms (*Ancylostoma duodenale* and *Necator americanus*) responsible for over 800 million and 740 million infections, respectively [1, 7, 8]. The global burden of STH infections is estimated at between 5 and 39 million disability-adjusted life years (DALYs), and in 2010, 5.18 million DALYs were estimated as associated with STH infections [9]. The greater burden of STH infections is found in the tropical countries including tropical South America, China, Southeast Asia, and Sub-Saharan Africa [5]. According to the WHO estimate, 42 countries in Africa are endemic for STH with 284 million cases occurring in both school aged and pre-school aged children. These children require periodic administration of preventive chemotherapy [1, 10].

In Ethiopia, the number of people living in STH endemic areas is estimated at 81 million, of which pre-school aged children account for 9.1 million [11]. The greatest numbers of intestinal worms are harbored in children resulting in diarrhea, loss of appetite, weight loss, growth retardation, malnutrition, anemia and cognitive defects [6, 7, 12–14].

In 2020, the World Health Organization (WHO) endorsed a new road map to combat NTDs including STH by 2030, and in fact substantial progress has been made in terms of controlling STH associated morbidity [3]. The WHO goal is to reduce the prevalence of moderate and heavy infection intensities with soil transmitted helminths among preschool and school aged children to below 2%, to make it no longer considered as public health problems by 2030 [3].

In line with WHO’s goal, Ethiopia has also set a similar goal to eliminate STH [11]. To achieve these goals in areas where prevalence of any soil-transmitted infection is 20% or higher, periodic mass administration of preventive chemotherapy (deworming) using annual or biannual single-dose albendazole or mebendazole is recommended by WHO for all pre-school and school aged children [15].

As part of the global actions towards Universal Health Coverage (UHC), ending NTDs is prioritized by 2030 in the Sustainable Development Goal (SDG) agenda under target 3.3 [3]. Moreover, working on NTDs helps the vision of universal health coverage, which means that all individuals and communities access the health services they need without suffering from financial suffering [16].

Since 2008 preventive chemotherapy (PC) against STH in PSAC has been implemented alongside Vitamin A distribution in the study area as well as at national level through community-based drug distribution platform [11]. In 2013, the first national master plan for NTDs was launched, and then the government of Ethiopia has been collaborating with the WHO and other partners for mapping all endemic districts to address SAC through the school-based mass drug administration [17]. Hence, we noted that PC was started before ten years ago to combat STH in PSAC at study area. However, the impact of deworming on STH infection status among PSAC has not been yet monitored and evaluated like SAC, and evidence is lacking at national level, particularly at the study area on prevalence, intensity and intervention status of STH infections among PSAC. Therefore, the present study aimed at to determine prevalence, intensity, and intervention status of STH infections among PSAC in Gamo Gofa zone, and to inform decision-making on STH controls and elimination programs.

## Methods

### Study area and period

This study was conducted in the former Gamo Gofa zone, Southern Ethiopia; in January 2019. The zone is found in Southern Nations, Nationalities, and Peoples’ Regional State (SNNPR), and it had 15 districts and two city administrations (CAs). All districts and CAs are endemic for STH, 15 had moderate prevalence and two had low prevalence. According to the 2007 estimate of Central Statistical Agency of Ethiopia, a total of 2,043,668 people live in the zone, of which 1,013,533 are males and 1,030,135 are females [18]. Arba Minch is the capital city of the zone, which is located at 435 km away from Addis Ababa, capital city of Ethiopia.

### Study design and population

A community-based cross-sectional study was conducted. The study population was all selected PSAC in the selected STH endemic *kebeles* (localities).

### Inclusion and exclusion criteria

Since there is no consistent definition of PSAC in the existing literatures, in this study, all children between 1 and 5 years who are not yet attending (primary) school were considered as pre-school children. PSAC who were unable to give stool samples at the time of data collection were excluded from the study. In addition, they were excluded in the event when they were seriously ill or care givers were unable to provide their information.

### Determining sample size and sampling technique

The sample size was determined using single population proportion formula,

$$n=\frac{{\left(Z_{\left(\frac{\alpha }{2}\right)}\right)}^{2}P(1-P)}{d^{2}};$$

we considered the followings elements to estimate a sample size which could represent the larger population: P, 25.7% (proportion of PSAC infected with STH) [19]; Z, 1.96 at significant level of alpha (α) of 0.05, and desired degree of precision (d) of 3%, and design effect of 3. The computed sample size was 2434, and by adding 10% non-response rate, the total computed sample size was 2678.

**Fig 1:**
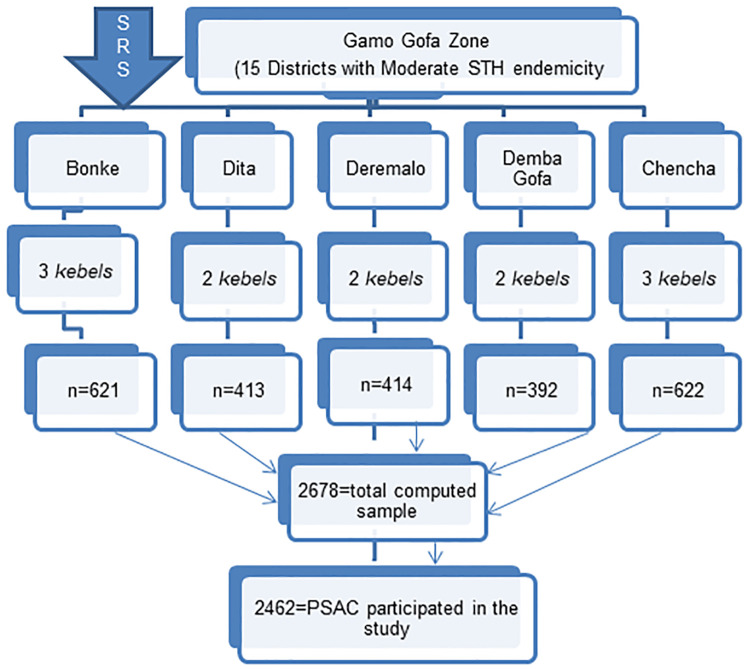
Sampling profile.

Multi-stage cluster sampling technique was employed in order to select study participants (Fig 1). First, districts and CAs STH endemicity status was identified based on findings of previous STH mapping survey conducted at national level (where 2 had low and 15 had moderate prevalence levels) [20]. We excluded 2 districts with low endemicity status since they were not eligible for preventive chemotherapy. Second, 5 districts (Chencha, Dita, Bonke, Deremalo and Demba Gofa) and 12 *kebeles* were selected from 15 districts using simple random sampling technique (SRS). Third, list of eligible households in each *kebeles* which had children between 1 and 5 years of age were identified by health extension workers (HEWs). Finally, one child from each household from each *kebeles* was selected by taking probability proportional-to-population size into account through consecutive home–to-home visit till the required sample size was obtained.

### Study variables and data collection

Variables included in this study were STH infection status (positive or negative for any STH), intensities of infections, socio-demographic and economic characteristics of parent or guardians, social determinants of health, wealth status of households and child related variables and implementation status of STH interventions. Data on these socio-demographic and other variables were collected through face-to-face interviews using pre-tested questionnaire from head of households (HHs) or mothers or guardians. Stool specimens were examined using the WHO recommended Kato-Katz technique under microscopy [21].

### Stool collection and processing

Fresh stool specimens were collected using clean, leak proof and screw cup container, and then the collected stool samples were transported in an icebox to nearby health facility for examination. The stool samples were processed within two hours of receipt or saved in an icebox where travel time exceeded two hours. Samples were examined in the local health center by Kato-Katz technique to determine the prevalence and intensities of STH infections.

### Quality control

Data quality was monitored through standard operational procedure, recruiting competent data collectors, pre-testing tools, training data collectors and supervisors, daily checking consistency and accuracy of collected data. The quality of data collection was closely monitored by supervisors. Stools were examined by qualified laboratory technicians. Duplicate slides were prepared per stool sample in order to ensure reliability. For the purpose of bench aid, pictures of parasites eggs were displayed on wall surface in front of microscopy examination for the purpose of internal quality control.

### Statistical analysis and measurements

First, data were edited and entered into EpiData 4.4.2, and then exported to SPSS software (IBM, version 25) for analysis. A difference in prevalence of STH between or among categories of variables was analyzed using Chi-Square test (X^2^). Summary statistics were computed, and data were presented with tables and graphs.

Wealth analysis was performed as, initially, reliability test was performed using the economic variables involved in measuring the wealth of the households. The variables which were used to compute the alpha value were entered into the principal component analysis. At the end of the principal component analysis, the wealth index was obtained as a continuous scale of relative wealth. Then, quintiles of the wealth index were created. Knowledge and practice on STH transmission and prevention were measured using 11 questions and score was computed by counting value within a case.

In this study, latrine cleanliness was stated as absence of faecal material or any dirt on the upper surface/floor of the latrine, and unsafe water was defined as untreated water obtained from well, river and spring, whereas safe water defined as water obtained from private or public tap water.

We calculated the prevalence by dividing the number of STH positive PSAC by the total number of participants. Intensity of STH infection is the number of helminths (worms) infecting an individual; for each parasite species it was analyzed as light, moderate and heavy infections based on number of eggs per gram of stool (EPG), and it was classified according to the WHO guidelines [22] as follow (Table 1).

**Table 1:** Criteria for classifying STH infection intensity for each species.

STH species	Intensity of infection (EPG)		
	Light	Moderate	Heavy
***A*. *lumbricoides***	(1–4999)	(5000–49999)	(≥50000)
***T*. *trichiura***	(1–999)	(1000–9999)	(≥10000)
**Hookworms**	(1–1999)	(2000–3999)	(≥4000)

According to WHO STH endemicity mapping classifications, there are three categories in line with implementation of mass drug administration (MDA): i) high transmission (where prevalence is >50%), ii) moderate transmission (where prevalence is between 20%-50%), and iii) low transmission (where prevalence is < 20%) [23, 24].

### Ethics statement

Ethical approval (reference number: CMHS/11222/111) was obtained from Institutional Research Ethics Review Board of Arba Minch University, College of Medicine and Health Sciences, Ethiopia. Oral and written consents were obtained from district administrators and heads of the households before survey was conducted. We obtained consent from parents or guardians as situations dictated. All children that tested positive for one or more STH were promptly treated with albendazole or mebendazole by health workers.

## Results

### Socio-demographic and economic characteristics

Details on socio-demographic and economic characteristics are presented in Table 2. A total of 2462 PSAC participated in this study. Of the total, 246 (10%) were under 2 years of age, and slightly more males participated than females (52% versus 48%). More than half of HHs (57.5%) did not attend any formal education (Table 2).

**Table 2:** Socio-demographic data of PSAC and HHs and economic characteristics (N = 2462).

Variable	Category	Frequency	Percent (%)
**Child sex**	Male	1281	52
	Female	1181	48
**Child age (years)**	<2 yrs.	246	10
	3–5 yrs.	2216	90
**Age of HH (years)**	<20	22	0.9
	20–29	404	16.4
	30–39	1284	52.1
	40–49	647	26.3
	55–59	59	2.4
	60 and above	46	1.9
**Sex of HH**	Male	2174	88.3
	Female	288	11.7
**Educational status**	No formal education	1416	57.5
	Elementary	508	20.6
	Secondary	279	11.3
	Diploma and above	259	10.5
**Occupation**	Farming	1606	65.2
	Employed	312	12.7
	Merchant	303	12.3
	Unemployed	78	3.2
	Daily laborer	163	6.6
**Family size**	<4	288	11.7
	4–6	1503	61.1
	7 and more	671	27.2
**Residence**	Urban	628	25.5
	Rural	1834	74.5
**Wealth quintile**	Wealthiest	488	19.8
	Wealthy	498	20.2
	Middle income	482	19.6
	Poor	498	20.2
	Poorest	496	20.2

### Prevalence of STH infections

**Fig 2:**
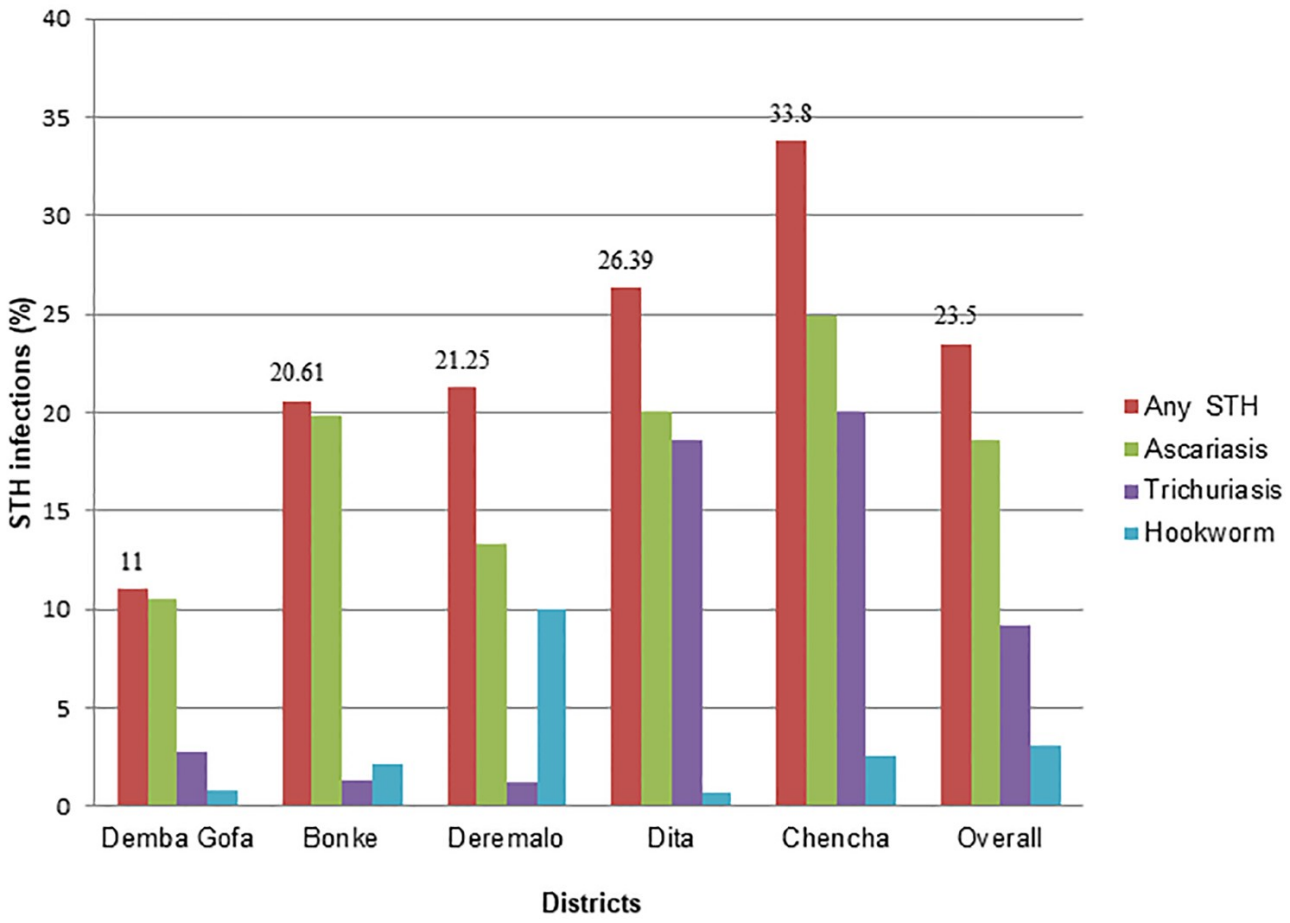
Prevalence of any STH infection dis-aggregated by districts among PSAC, Gamo Gofa zone, Southern Ethiopia, 2019 (N = 2462).

Of the total surveyed children, 23.5% (578/2462) (95% confidence interval (CI) = 21.8%–25.2%) had at least one type of STH infection. Ascariasis was the most prevalent (18.6%), followed by trichiurasis (9.2%) and hookworms (3.1%). Mixed STH infections (*Ascaris lumbricoides* and *Trichuris trichiura*) found in 7.4% of PSAC. The highest prevalence (33.8%) of any one of STH infection observed in Chencha district, as contrasted to the lowest prevalence (11%) found in Demba Gofa district. In Deremalo district, considerable amount of hookworm infections (10%) were revealed (Fig 2).

The prevalence of STH infections was slightly higher at ≤2 years, while comparing across the age-group (1–5 years). On the other hand, prevalence of any STH infections among females (24.3%) was a little higher than the male’s (22.7%), and a higher prevalence of STH infections (24.6%) in rural area was noticed than urban (20.1%) (Table 3).

**Table 3:** Prevalence of STH infections among PSAC dis-aggregated by different selected variables in Gamo Gofa zone, 2019 (N = 2462).

Variable	Category	STH Infection status		X^2^-test	P-value
		Negative, n (%)	Positive, n (%)		
**Child sex**	Male	990 (77.3)	291 (22.7)	0.354	0.366
	Female	894 (75.7)	287 (24.3)		
**Child age group (years)**	≤2 yrs.	187 (76)	59 (24)	0.039	0.843
	3–5 yrs.	1697 (76.6)	519 (23.4)		
**Place of residence**	Urban	502 (79.9)	126 (20.1)	**5.467**	**0.019**
	Rural	1382 (75.4)	452 (24.6)		
**Child age (years)**	1	187 (76)	59 (24)	0.914	0.923
	2	414 (75.1)	137 (24.9)		
	3	563 (77)	168 (23)		
	4	522 (77.2)	154 (22.8)		
	5	198 (76.7)	60 (23.3)		
**Child soil eating habit**	No	1518 (76.9)	457 (23.1)	0.371	0.542
	Yes	261 (78.4)	72 (21.6)		
**Family size**	<4	220 (76.4)	68 (23.6)	2.219	0.330
	4–6	1164 (77.4)	339 (22.6)		
	7 and above	500 (74.5)	171 (25.5)		
**Mothers’ (guardians’) educational status**	Can’t read and write	774 (76.9)	233 (23.1)	5.432	0.246
	Can read and write	291 (73.9)	103 (26.1)		
	Elementary	486 (76.1)	153 (23.9)		
	Secondary	225 (76.8)	68 (23.2)		
	Diploma and above	108 (83.7)	21 (16.3)		
**Mothers’ (guardians’) occupation**	Farming	974 (74.6)	331 (25.4)	**16.288**	**0.003**
	Employed (Gov.)	166 (81.8)	37 (18.2)		
	Merchant	348 (73.7)	124 (26.3)		
	Unemployed	334 (82.3)	72 (17.7)		
	Others*	62 (81.6)	14 (18.4)		
**Wealth quintile**	Highest	379 (77.7)	109 (22.3)	1.372	0.712
	Fourth	379 (76.1)	119 (23.9)		
	Middle income	374 (77.6)	108 (22.4)		
	Second	369 (74.1)	129 (25.9)		
	Lowest	383 (77.2)	113 (22.8)		
**Water source**	Pipe	891 (79)	237 (21)	**8.289**	**0.04**
	Well	223 (76.1)	70 (23.9)		
	Public bono	721 (74.3)	250 (25.7)		
	Other**	49 (70)	21 (30)		

* = Daily laborer and housewife

** = River and spring

### STH infections intensity

In majority of STH infections (85%), low infection intensities are associated with hookworms and *Trichuris trichiura* infections, while 15.1% of ascariasis had moderate infection intensity (Table 4). All of the moderate infection intensities were from Chencha and Bonke districts.

**Table 4:** STH infection intensity among PSAC in Gamo Gofa zone, Southern Ethiopia, 2019 (N = 2462).

Type of STH infection	Mean (EPG)	Infection intensity		
		Light	Moderate	Total infected PSAC
**Ascariasis**	2152	388 (84.90)	69 (15.1)	457
**Hookworms**	154	76 (100)	0	76
**Trichiurasis**	135	226 (100)	0	226

### Intervention status against STH infections

#### I. Preventive chemotherapy coverage against STH

The overall self-reported treatment coverage with albendazole (ALB) against STH among PSAC was 68.7% (1691/2462) in the last year before the survey.

#### II. Knowledge and practice (KP) of mothers or guardians related to STH transmission and prevention

Table 5 presents the details on KP on STH transmission and prevention among mothers or guardians. Of the total surveyed mothers or guardians, almost 93% (2283/2462) achieved less than the mean KP score (≤5) on prevention of STH, 42.7% (1052/2462) did not wash hand after defecation, and 77.7% (1913/2462) got information on STH from health extension workers (HEWs) (Table 5).

**Table 5:** Knowledge and practice (KP) of mothers or guardians of PSAC related to STH transmission and prevention in Gamo Gofa zone, Southern Ethiopia, 2019 (N = 2462).

Variables	Category	Frequency	%
**Knew about STH**	Yes	2208	89.7
	No	254	10.3
**Knew at least one STH transmission (n = 2208)**	Yes	2153	97.5
	No	55	2.5
**Knew at least one STH prevention way (n = 2208)**	Yes	2135	96.7
	No	73	13.5
**KP score on STH transmission**	≤5	2262	91.9
	>5	200	8.1
**KP score on STH prevention**	≤5	2283	92.7
	>5	179	7.3
**Source of information about STH**	Health facility	271	11
	HEWs	1913	77.7
	Radio or TV	24	1
	None	254	10.3
**Habit of washing hand after latrine**	Yes	1410	57.3
	No	1052	42.7
**Habit of washing hand before meal**	Yes	2347	95.3
	No	2462	4.7
**Habit of washing hand after cleaning child**	Yes	2229	90.5
	No	233	9.5
**Habit of washing hand before cooking**	Yes	2069	84
	No	393	16
**Habit of washing fruits or vegetables before eating**	Yes	1841	74.8
	No	621	25.2
**Habit of washing hand after work**	Yes	2253	91.5
	No	209	8.5
**Where do you dispose child’s faeces?**	Within home compound	194	7.9
	Toilet	2002	81.3
	Garbage	266	10.8

#### III. Water, sanitation and hygiene (WASH)

Table 6 presents the details on WASH characteristics at HHs level. This study also revealed households data as, 39.4% used water from hand-dug well; 52.5% of need to travel more than 30 minutes to collect water; 77.5% did not use treated water, and 48.9% had no hand washing facility (Table 6).

**Table 6:** WASH characteristics of households among PSAC participants in Gamo Gofa zone, Southern Ethiopia, 2019.

Variables	Category	Frequency	%
**Source of water**	Pipe	1128	45.8
	Well	293	11.9
	Public bono	971	39.5
	Other*	70	2.8
**Distance from water source**	< 30 min	1170	47.5
	≥ 30 min	1292	52.5
**Adequate water**	No	536	21.8
	Yes	1926	78.2
**Habit of treating water**	No	1909	77.5
	Yes	553	22.5
**Do you have latrine**	Yes	2397	97.4
	No	65	2.6
**Type of latrine (n = 2397)**	Pit	2360	98.5
	Improved pit latrine	37	1.5
**Latrine clean**	No	1766	73.7
	Yes	631	26.3
**Hand washing facility around toilet (functional)**	No	1225	51.1
	Yes	1172	48.9
**Soap or ash available at hand washing station (n = 1172)**	Yes	298	25.4
	No	874	74.6
**Reason for absence of latrine (n = 65)**	No space	6	9.2
	No money	10	15.4
	No skill	9	13.8
	Did not know importance	40	61.5
**If no latrine, where do you defecate (n = 65)**	Open field	56	86.2
	Public	9	13.8

* = river and spring

## Discussion

This study showed operational context specific evidences on prevalence, intensity and intervention status of soil-transmitted helminth infections among PSAC. It is noted that a significant proportion of PSAC are suffering from STH infections despite provision of mass drug administration at the study area. Also, gaps in the intervention status (PC coverage, WASH and KP on STH prevention) against STH were highlighted, which need to be addressed by the STH programs.

In this study, the overall prevalence of STH infections with at least one STH parasite was 23.5%, which would be classified into the moderate transmission category (where prevalence is between 20% and 50%), and qualifies the requirement of annual STH mass drug administration [24]. Ascariasis was the most prevalent infection (18.6%), followed by trichiurasis (9.2%) and hookworms (3.1%). Most of the positive cases with STH were showed low infection intensities; which could be the positive impact of PC on morbidity reduction. However, 15.1% cases of ascariasis were revealed the moderate infection intensities; This result is much higher than WHO elimination target of STH (which is defined as < 2% proportion of soil-transmitted helminth infections of moderate and heavy intensity due to *A*. *lumbricoides*, *T*. *trichuria*, *N*. *americanus and A*. *duodenal*) [3]. In our study, despite initiation of preventive chemotherapy about 10 years ago, the prevalence of STH did not show significant reduction probably due to weak implementation of control strategies-social behavioral change communication (SBCC), inadequate mass drug administration coverage and WASH, as highlighted in the findings of this study.

Although the overall prevalence of STH infections observed in this study is comparable with studies conducted in another parts of Ethiopia (Butajira and Wonji) and West China [25–27], our study reached out larger area and powered with sufficient sample size to assess prevalence, intensity and intervention status of STH among PSAC. On the contrary, the STH prevalence in our study is slightly lower than the prevalence reported in Dembiya, northwest Ethiopia [19]. In addition, the prevalence in our study is significantly lower than the findings of other studies conducted in another part of Ethiopia and in Nigeria, Cameroon, Ecuador, Uganda, Kenya and Honduras [28–35]. These differences observed from our study could be due to variation in socio-cultural, social determinants, behavioral characteristics and implementation of prevention and control measures.

In our study, ascariasis was identified as the commonest species of STH, and this finding supports studies done in another part of Ethiopia, Nigeria and China [19, 25, 27, 28]. On the contrary, some other studies conducted in Ethiopia, Ecuador and Honduras showed high prevalence of Trichiurasis [31, 34, 36], and a study conducted in Uganda showed high prevalence of hookworm [32]. These differences might be related to variation in environmental factors, such as climate, rainfall, topography, surface temperature, altitude, and soil type which have a great impact on the distribution of STH [37]. Moreover, in this study we found significant amounts of mixed infections, 7.4% of PSAC were infected with two STH species (*Ascaris lumbricodes and Trichuris trichiura*); this finding is in line with a study conducted in another areas of Ethiopia and Nigeria [25, 26, 28].

In this study, slightly higher prevalence at age ≤2 years was observed, while comparing across the age-group (1–5 years); the possible explanation related to this difference is due to the fact that current mass drug administration among PSAC often does not include children age ≤2 years. On the contrary, other studies revealed numerical increase in prevalence of STH as age increase [25, 28]. In addition, in this study, prevalence of any STH infection among females (24.3%) was a little higher than the male’s prevalence (22.7%), this result supports the findings of a study conducted in another part of Ethiopia [25]. These differences might be due to low access and uptake of preventive chemotherapy among females, as justified by data of our study.

In addition, significant proportion (15.1%) of moderate intensity ascariasis was observed in our study, and this finding is higher than results of a study conducted in Butajira, Ethiopia and Honduras [25, 34]. This finding is much higher than WHO elimination target of STH (which is defined as <2% proportion of soil-transmitted helminth infections of moderate and heavy intensities due to *A*. *lumbricoides*, *T*. *trichuria*, *N*. *americanus and A*. *duodenal*) [3]. The possible explanation related to this difference could be consistency and frequency of mass drug administration that may affect intensity of infections [23].

Furthermore, in our study, gaps in the intervention against STH among PSAC were highlighted. The treatment coverage of ALB in this survey against STH among PSAC was (68.7%); which is lower than the national coverage (71%) of Ethiopia and WHO’s target (minimum of 75%) [15, 38]. The possible explanation for the unmet target of PC coverage could be driven by low knowledge of the community regarding the benefits of PC on STH prevention. Most importantly, study participants in this study were pre-school age children, who might not be reached out by the deworming program, especially those ≤ 2 years old. By taking the significant burden of STH among children ≤2 (1–2 years) into consideration, the community based deworming program should reach out these children in collaboration with the community and other stakeholders. On the other hand, obviously, school age children could have better chance to be reached by school-based deworming.

Although site-specific data are required in our case, the importance of WASH interventions to control and eliminate STH reported in different studies [39–41]. However, the status of WASH interventions observed in our study is much lower than WHO’s targets that are supposed to be achieved by 2030 [3]. In this study, inadequate WASH infrastructures were observed at household level, where 39.4% were using water from hand-dug well; 52.5% were walking more than 30 minutes to collect water; 77.5% did not treat water, and 48.9% of had no hand washing facility. Of the total surveyed mothers or guardians, almost 93% achieved less than the mean KP score (≤5) on prevention of STH, 42.7% did not wash hand after defecation, and 77.7% got information on STH from health extension workers. The possible reason for these findings could be related to weak and inconsistent social behavioral change communication (SBCC) intervention.

The most outstanding strength of our study is that it is addressing an important national operational research priority with large sample size; which is focusing on parasitological monitoring and control strategies of STH among pre-school aged children.

In this study, there are limitations that need to be taken into account. There might be underestimation of prevalence of STH due to the fact that (1) we collected single stool specimen, which could cause variation in eggs excretion over different times (hours) within a day and across different days; (2) samples were collected from remote villages and there might be rapid desiccation of hookworm eggs in the stool samples, and (3) even though the Kato–Katz technique is sensitive in detecting moderate and high infection intensities (MHI), it has lower detection power, and therefore lower positive predictive values in low-prevalence settings [27, 42].

## Conclusions

Data from our study showed that substantive proportion of pre-school age children in the study area are suffering from STH infections despite provision of preventive chemotherapy distribution at the study area. Also, gaps in the intervention (PC coverage, WASH and KP on STH prevention) to control and eliminate STH were highlighted. Thus, a call for action is demanding to address those gaps, and impact of the interventions should be monitor regularly to achieve the national goal of STH elimination in Ethiopia by 2030. Further, operational research focusing on implementation of PC and impact of specific WASH factors needs to be conducted in different transmission (high, moderate and low) settings to determine more precise epidemiological, environmental and host factors and strengthen STH control and elimination efforts.

## Supporting information

S1 Questionnaire (DOCX)

## References

[1] Montresor A, Trouleau W, Mupfasoni D, Bangert M, Joseph SA, Mikhailov A, et al Preventive chemotherapy to control soil-transmitted helminthiasis averted more than 500 000 DALYs in 2015. Transactions of the Royal Society of Tropical Medicine and Hygiene. 2017 10 1; 111(10):457–63. 10.1093/trstmh/trx082 29346640 PMC5808863

[2] Hotez PJ, Fenwick A, Savioli L, Molyneux DH. Rescuing the bottom billion through control of neglected tropical diseases. The Lancet. 2009 5 2; 373(9674):1570–5. 10.1016/S0140-6736(09)60233-6 19410718

[3] World Health Organization. Ending the neglect to attain the sustainable development goals: A road map for neglected tropical diseases 2021–2030. World Health Organization; 2020.

[4] De Silva NR, Brooker S, Hotez PJ, Montresor A, Engels D, Savioli L. Soil-transmitted helminth infections: updating the global picture. Trends in Parasitology. 2003 12 1; 19(12):547–51. 10.1016/j.pt.2003.10.002 14642761

[5] Bethony J, Brooker S, Albonico M, Geiger SM, Loukas A, Diemert D, et al Soil-transmitted helminth infections: ascariasis, trichuriasis, and hookworm. The Lancet. 2006 5 6; 367(9521):1521–32. 10.1016/S0140-6736(06)68653-4 16679166

[6] Albonico M, Allen H, Chitsulo L, Engels D, Gabrielli AF, Savioli L. Controlling soil-transmitted helminthiasis in pre-school-age children through preventive chemotherapy. PLoS Neglected Tropical Diseases. 2008 3; 2(3). 10.1371/journal.pntd.0000126 18365031 PMC2274864

[7] Schistosomiasis WH. soil-transmitted helminth infections–preliminary estimates of the number of children treated with albendazole or mebendazole. Wkly Epidemiol Rec. 2006 4 21; 81(16):145–64. 16673507

[8] Pullan RL, Brooker SJ. The global limits and population at risk of soil-transmitted helminth infections in 2010. Parasites & Vectors. 2012 12 1; 5(1):81 10.1186/1756-3305-5-81 22537799 PMC3419672

[9] Brooker S. Estimating the global distribution and disease burden of intestinal nematode infections: adding up the numbers–a review. International Journal for Parasitology. 2010 8 15; 40(10):1137–44. 10.1016/j.ijpara.2010.04.004 20430032 PMC3034165

[10] World Health Organization. Intestinal worms: Soil-transmitted helminthes World Health Organization; 2011.

[11] Federal Democratic Republic of Ethiopia Ministry of Health. Second Edition of Ethiopia National Master Plan for Neglected Tropical Diseases. Addis Ababa, Ethiopia.2016

[12] Tchuenté LA. Control of schistosomiasis and soil-transmitted helminthiasis in sub-Saharan Africa: challenges and prospects. Current Topics in Tropical Medicine. 2012 3 16:359–76.

[13] Stephenson LS, Latham MC, Ottesen EA. Malnutrition and parasitic helminth infections. Parasitology. 2000 10; 121(S1):S23–38. 10.1017/s0031182000006491 11386688

[14] Soeripto N. Reinfection and infection rates of soil-transmitted-helminths in Kemiri Sewu, Yogyakarta, Indonesia. Southeast Asian J Trop Med Public Health. 1991 6; 22(2):216–21. 1948282

[15] World Health Organization. Guideline: preventive chemotherapy to control soil-transmitted 381 helminth infections in at-risk population groups. World Health Organization; 2017. 29578660

[16] World Health Organization. Universal health coverage WHO fact sheet. Geneva World Health Organization; 2019.

[17] Federal Democratic Republic of Ethiopia Ministry of Health. Ethiopian National Master Plan for Neglected Tropical Diseases (2013–2015). Addis Ababa, Ethiopia; 2013

[18] Central statistical agency (2013) Population Projection for Ethiopia 2007–2037. Addis Ababa, Ethiopia.

[19] Gizaw Z, Adane T, Azanaw J, Addisu A, Haile D. Childhood intestinal parasitic infection and sanitation predictors in rural Dembiya, northwest Ethiopia. Environmental health and preventive medicine. 2018 12; 23(1):26 10.1186/s12199-018-0714-3 29933747 PMC6015452

[20] Leta GT, Mekete K, Wuletaw Y, Gebretsadik A, Sime H, Mekasha S, et al. National mapping of soil-transmitted helminth and schistosome infections in Ethiopia. 10.1186/s13071-020-04317-6 PMC7466696 32873333

[21] World Health Organization. Kato-Katz technique: Bench Aids for the Diagnosis of Intestinal Paraistes. 1994.

[22] WHO Expert Committee. Prevention and control of schistosomiasis and soil-transmitted helminthiasis World Health Organization technical report series. 2002; 912: i 12592987

[23] World Health Organization. Preventive chemotherapy in human helminthiasis Coordinated use of anthelminthic drugs in control interventions: a manual for health professionals and programme managers. World Health Organization; 2006.

[24] World Health Organization. Soil-transmitted helminthiases: eliminating as public health problem soil-transmitted helminthiases in children: progress report 2001–2010 and strategic plan 2011–2020.

[25] Shumbej T, Belay T, Mekonnen Z, Tefera T, Zemene E. Soil-transmitted helminths and associated factors among pre-school children in Butajira Town, South-Central Ethiopia: a community-based cross-sectional study. PloS one. 2015; 10(8). 10.1371/journal.pone.0136342 26305361 PMC4548951

[26] Degarege A, Erko B. Prevalence of intestinal parasitic infections among children under five years of age with emphasis on Schistosoma mansoni in Wonji Shoa Sugar Estate, Ethiopia. PloS one. 2014 10 8; 9(10):e109793 10.1371/journal.pone.0109793 25296337 PMC4190315

[27] Wang X, Zhang L, Luo R, Wang G, Chen Y, Medina A, et al Soil-transmitted helminth infections and correlated risk factors in preschool and school-aged children in rural southwest China. PLoS One. 2012; 7(9). 10.1371/journal.pone.0045939 23029330 PMC3459941

[28] Sowemimo OA, Asaolu SO. Current status of soil-transmitted helminthiases among pre-school and school-aged children from Ile-Ife, Osun State, Nigeria. Journal of Helminthology. 2011 9; 85(3):234–8. 10.1017/S0022149X10000489 20810012

[29] Nkengazong L, Njiokou F, Wanji S, Teukeng F, Enyong P, Asonganyi T. Prevalence of soil transmitted helminths and impact of Albendazole on parasitic indices in Kotto Barombi and Marumba II villages (South-West Cameroon). African Journal of Environmental Science and Technology. 2010; 4(3).

[30] Dana D, Mekonnen Z, Emana D, Ayana M, Getachew M, Workneh N, et al Prevalence and intensity of soil-transmitted helminth infections among pre-school age children in 12 kindergartens in Jimma Town, southwest Ethiopia. Transactions of the Royal Society of Tropical Medicine and Hygiene. 2015 3 1; 109(3):225–7. 10.1093/trstmh/tru178 25371496

[31] Moncayo AL, Lovato R, Cooper PJ. Soil-transmitted helminth infections and nutritional status in Ecuador: findings from a national survey and implications for control strategies. BMJ open. 2018 4 1; 8(4):e021319 10.1136/bmjopen-2017-021319 29705768 PMC5931300

[32] Ojja S, Kisaka S, Ediau M, Tuhebwe D, Kisakye AN, Halage AA, et al Prevalence, intensity and factors associated with soil-transmitted helminths infections among preschool-age children in Hoima district, rural western Uganda. BMC infectious diseases. 2018 12 1; 18(1):408 10.1186/s12879-018-3289-0 30119650 PMC6098587

[33] Davis SM, Worrell CM, Wiegand RE, Odero KO, Suchdev PS, Ruth LJ, et al Soil-transmitted helminths in pre-school-aged and school-aged children in an urban slum: a cross-sectional study of prevalence, distribution, and associated exposures. The American Journal of Tropical Medicine and Hygiene. 2014 11 5; 91(5):1002–10. 10.4269/ajtmh.14-0060 25157123 PMC4228865

[34] Torres RE, Garcia DN, Sandoval GA, Santana AH, Singh P, Bucheli ST, et al Prevalence and intensity of soil-transmitted helminthiasis, prevalence of malaria and nutritional status of school going children in Honduras. PLoS Neglected Tropical Diseases. 2014 10; 8(10). 10.1371/journal.pntd.0003248 PMC4199552 25330010

[35] Tekalign E, Bajiro M, Ayana M, Tiruneh A, Belay T. Prevalence and Intensity of Soil-Transmitted Helminth Infection among Rural Community of Southwest Ethiopia: A Community-Based Study. BioMed Research International. 2019; 2019 10.1155/2019/3687873 31915688 PMC6931019

[36] Nyantekyi LA, Legesse M, Belay M, Tadesse K, Manaye K, Macias C, et al Intestinal parasitic infections among under-five children and maternal awareness about the infections in Shesha Kekele, Wondo Genet, Southern Ethiopia. Ethiopian Journal of Health Development. 2010; 24(3).

[37] Hotez PJ, Brindley PJ, Bethony JM, King CH, Pearce EJ, Jacobson J. Helminth infections: the great neglected tropical diseases. The Journal of Clinical Investigation. 2008 4 1; 118(4):1311–21. 10.1172/JCI34261 18382743 PMC2276811

[38] Asfaw MA, Zerdo Z, Churko C, Seife F, Yihune M, Chisha Y, et al Preventive chemotherapy coverage against soil-transmitted helminth infection among school age children: Implications from coverage validation survey in Ethiopia, 2019. Plos one. 2020 6 26; 15(6):e0235281 10.1371/journal.pone.0235281 32589660 PMC7319348

[39] Freeman MC, Ogden S, Jacobson J, Abbott D, Addiss DG, Amnie AG, et al Integration of water, sanitation, and hygiene for the prevention and control of neglected tropical diseases: a rationale for inter-sectoral collaboration. PLoS Neglected Tropical Diseases. 2013 9; 7(9). 10.1371/journal.pntd.0002439 PMC3784463 24086781

[40] Ziegelbauer K, Speich B, Mäusezahl D, Bos R, Keiser J, Utzinger J. Effect of sanitation on soil-transmitted helminth infection: systematic review and meta-analysis. PLoS Med. 2012 1 24; 9(1):e1001162 10.1371/journal.pmed.1001162 22291577 PMC3265535

[41] Becker SL, Liwanag HJ, Snyder JS, Akogun O, Belizario V Jr, Freeman MC, et al Toward the 2020 goal of soil-transmitted helminthiasis control and elimination. PLoS Neglected Tropical Diseases. 2018 8; 12(8). 10.1371/journal.pntd.0006606 30106975 PMC6091919

[42] Freeman MC, Akogun O, Belizario V Jr, Brooker SJ, Gyorkos TW, Imtiaz R, et al Challenges and opportunities for control and elimination of soil-transmitted helminth infection beyond 2020. PLoS Neglected Tropical Diseases. 2019 4; 13(4). 10.1371/journal.pntd.0007201 30973872 PMC6459486

