# Supplementary material for: Soil-transmitted helminth infections among pre-school aged children in Gamo Gofa zone, Southern Ethiopia: Prevalence, intensity and intervention status
Source: PLoS One. 2020 Dec 15;15(12):e0243946. doi: 10.1371/journal.pone.0243946 (PMC7737900; doi:10.1371/journal.pone.0243946)
Supplement: S1 Questionnaire — (DOCX) [file pone.0243946.s001.docx]

**S1 Questionnaire and Kato-Katz technique**

**Part one: Socio-demographic and economic characteristics**

***Kebele*______________________; House number ______________; Child ID______**

| **Serial number** | | **Questions** | **Option** | |
| --- | --- | --- | --- | --- |
| **Socio-demographic characteristics** | | | | |
| 101 | | Sex of head of households (HHs) | 1.Male 2.Female | |
| 102 | | How old are you, HHs? | ______ (years) | |
| 103 | | Where is your place of residence? | 1.Urban 2.Rural | |
| 104 | | What is your current marital status, HHs? | 1.Married 2.Unmarried/single  3. Separated 4. Windowed  5.Others(specify)_________ | |
| 105 | | What is the highest educational level you achieved, HHs? | 1.Can’t read and write  2.Can read write  3.Grade1-8  4.Grade 9-12  5.College and above | |
| 106 | | What is your occupation, HHs? | 1.Farmer  2.Employed (government)  3.Business (Self-employed)  4.Unemployed  5.Other (specify) _________ | |
| 107 | | Mothers’ (guardians’) educational status | 1.Can’t read and write  2.Can read write  3.Grade1-8  4.Grade 9-12  5.College and above | |
| 108 | | Mothers’ (guardians’) occupation | 1.Farmer  2.Employed (government)  3.Business (Self-employed)  4. Housewife  5.Unemployed  6.Other (specify) _________ | |
| 109 | | How many individuals live in the household? | ______ (in number) | |
| **Economic characteristics of household** (1. Yes 0. No) | | | | |
| 110 | Is there electricity in home? | | 1. 0 | |
| 111 | Does the household currently have any of the following animals?(circle the answer) | | | |
|  | Oxen | | 1 | 0 |
|  | Cow | | 1 | 0 |
|  | Goat | | 1 | 0 |
|  | Sheep | | 1 | 0 |
|  | Chicken | | 1 | 0 |
| 112 | Does the household currently have any of the following items? (circle the answer) **(1. Yes 0. No)** | | | |
|  | Functioning radio | | 1 | 0 |
|  | Functioning television | | 1 | 0 |
|  | Functioning tape recorder/CD player | | 1 | 0 |
|  | Kerosene stove | | 1 | 0 |
|  | Telephone | | 1 | 0 |
|  | Electric stove? | | 1 | 0 |
|  | Sofa | | 1 | 0 |
|  | Bed | | 1 | 0 |
|  | Spring mattress | | 1 | 0 |
|  | Foam mattress | | 1 | 0 |
|  | Grass mattress | | 1 | 0 |
|  | Chair | | 1 | 0 |
|  | Table | | 1 | 0 |
|  | Bicycle | | 1 | 0 |
|  | Telephone | | 1 | 0 |

**Part two: Child characteristics and deworming (ask information from primary caregivers)**

| **Serial number** | **Questions** | **Option** |
| --- | --- | --- |
| 201 | Sex of child | 1.Male 2.Female |
| 202 | Age of child | _____________ (years) |
| 203 | Has your child habit of eating soil? | 1. Yes 2. No |
| 204 | Did your child receive deworming drug in last year? | 1 Yes 2. No |
| 205 | Where do you wash child’s body? | 1. Home 2. River 3. Other (specify) _________ |
| 206 | Does your child have habit of move on barefoot? | 1. Yes 2. No |

**Part three: Knowledge and practice of mothers/caregivers related to transmission & prevention of STH (ask information from primary caregivers)**

| **Serial number** | **Questions** | **Option** |
| --- | --- | --- |
| 301 | Do you know about STH infection? | 1. Yes 2. No |
| 302 | Sources of information related to STH (one answer is possible) | 1. Health facility 2. Community health worker  3. Radio/TV, talk in the community  4. No information |
| 303 | How STH is transmitted? (don’t read the options and circle the mentioned) | - Contaminated foods (1. Yes 0. No) - Contaminated water (1. Yes 0. No) - Not washing hands before cooking foods (1. Yes 0. No) - Not washing hands before eating foods (1. Yes 0. No) - Not washing hands after toilet (1. Yes 0. No) - Not washing hands before feeding children (1. Yes 0. No) - Soil eating habit of child (1. Yes 0. No) - Not washing fruit and vegetables before eating (1. Yes 0. No) - Untrimmed and long nail (1. Yes 0. No) - Eating raw foods (1. Yes 0. No) - Not using toilet (1. Yes 0. No) |
| 304 | How STH is prevented and do you practice?  ( don’t read the options) (circle the mentioned) | - Food hygiene (1. Yes 0. No) - Having safe water (1. Yes 0. No) - Washing hands before cooking foods   (1. Yes 0. No)   - Washing hands before eating foods   (1. Yes 0. No)   - Washing hands after toilet (1. Yes 0. No) - Washing hands before feeding children   (1. Yes 0. No)   - Soil eating habit (1. Yes 0. No) - Washing fruit and vegetables before eating (1. Yes 0. No) - Having trimmed nail (1. Yes 0. No) - Not eating raw foods (1. Yes 0. No) - Using toilet (1. Yes 0. No) - Treating infected individuals (1. Yes 0. No) |

**Part four: Water, Sanitation and Hygiene (WASH) characteristics of households (ask information from primary caregivers)**

| **Serial number** | **Questions** | **Option** |
| --- | --- | --- |
| 401 | What is your main source of drinking water? | 1.Private tap  2. Public tap  3. Well  4.Other (specify)_________ |
| 402 | Do you obtain sufficient amount of water for household consumption? | 1. Yes 2. No |
| 403 | Do you treat water before drinking | 1.Yes  2.No (if no skip to q406) |
| 404 | How do you treat water? | 1. Boiling 2. Chemical (agar)  3. Both |
| 405 | How often do you treat water? | 1. Always 2. Usually  3. Sometimes 4. Never |
| 406 | How far the water source from home? | ____ (minutes) |
| 407 | Do you have latrine? | 1.Yes 2.No (if no skip to q413) |
| 408 | Which type of latrine you have? | 1. Simple pit 2. Ventilated improved pit latrine (VIP)  3. Pour flush 4. Other (specify) |
| 409 | Is the latrine clean? (either dirt or faeces not observed on latrine floor) | 1.Yes 2.No |
| 410 | Functional hand washing station available around toilet? | 1.Yes 2.No |
| 411 | Do you wash your hand after latrine? | 1.Yes 2.No |
| 412 | Is there soap or ash at hand washing station? | 1.Yes 2.No |
| 413 | What is the reason for not to having latrine? | 1.Have no place  2. No money  3.legal permission is difficult  4. Lack of knowledge  5. Other____________ |
| 414 | Where do you defecate? | 1.Open field  2. By payment in hotels  3. Other (specify)___________ |
| 415 | Where do you dispose child’s feces? | 1. Anywhere in the compound  2. In toilet  3. In garbage box  4. Other (specify) _________ |
| 416 | Do you wash your hands before cooking foods? | 1. Yes 2. No |
| 417 | Do you wash your hands after working? | 1. Yes 2. No |
| 418 | Do you wash raw fruits or vegetables before eating? | 1. Yes 2. No |
| 419 | Do you wash your hands before eating foods? | 1.Yes 2.No |
| 420 | Do you wash your hands after cleaning your child? | 1.Yes 2.No |

**Part five: Kato-Katz technique (laboratory procedure) (WHO, 1994)**

1. A small amount of stool sample was first pressed through a sieve to remove large particles.
2. Part of the sieved stool was then transferred to the hole of a template on a slide using flat-sided spatula.
3. The hole was filled after which the template was removed and the remaining sieved sample was covered with cellophane which had been pre-soaked in glycerol.
4. Then, the microscope slide was inverted and firmly pressed the fecal sample against the hydrophilic cellophane strip on another microscope slide or on a smooth hard surface.
5. The fecal material was spread evenly between the microscope slide and the cellophane strip; it should be possible to read newspaper print through the smear after clarification.
6. The slide was carefully removed by gently sliding it sideways to avoid separating the cellophane strip or lifting it off.
7. Then, the slide was placed on the bench with the cellophane upwards, and water evaporates while glycerol clears the smear.
8. Finally, the smears were examined in a systematic manner and the number of eggs of each species reported.
9. Later multiplied by 24 (for a 41.7 mg template) to give the number of eggs per gram of stool.
