# Supplementary material for: Soil-transmitted helminth infections among pre-school aged children in Gamo Gofa zone, Southern Ethiopia: Prevalence, intensity and intervention status
Source: PLoS One. 2020 Dec 15;15(12):e0243946. doi: 10.1371/journal.pone.0243946 (PMC7737900; doi:10.1371/journal.pone.0243946)

PLOS ONE

Soil-transmitted helminth infections among pre-school aged children in Gamo Gofa zone, Southern Ethiopia: Prevalence, intensity and intervention status

--Manuscript Draft--

**Manuscript Number:**	PONE-D-20-25387
**Article Type:**	Research Article
**Full Title:**	Soil-transmitted helminth infections among pre-school aged children in Gamo Gofa zone, Southern Ethiopia: Prevalence, intensity and intervention status
**Short Title:**	Soil-transmitted helminth infections and intervention status among pre-school ag[illegible] children in Gamo Gofa zone
**Corresponding Author:**	Mekuria Asnakew Asfaw<br>Arba Minch University<br>Arba Minch, ETHIOPIA
**Keywords:**	Soil-transmitted helminth; Neglected tropical diseases, Pre-school age children; Kato-Katz technique; Prevalence; Intensity, Control strategies of STH; Operational research
**Abstract:**	Background<br>Soil-transmitted helminths (STH), i.e., Ascaris lumbricoides, Trichuris trichiura and hookworms are among the most prevalent neglected tropical diseases (NTDs) in Ethiopia. Although pre-school age children (PSAC) pay high morbidity toll associated with STH infections, evidence on prevalence, intensity and intervention status is lacking in Ethiopia. This study, therefore, aimed to address that missing knowledge gap.<br>Methods<br>We did a community-based cross-sectional study in five districts of Gamo Gofa zone, southern Ethiopia; from December 2018 to January 2019. Data were collected using pre-tested questionnaire, and the Kato-Katz technique was used to diagnose parasites egg in stool. Then, the collected data were edited and entered into EpiData 4.4.2, and exported to SPSS software (IBM, version 25) for analysis.<br>Results<br>A total of 2462 PSAC participated in this study. Overall, the prevalence of STH was 23.5% (578/2462) (95% confidence interval (CI) = 21.8–25.2%) with highest prevalence revealed in Chencha district (33.8%). Ascaris lumbricoides was the most prevalent (18.6%), followed by Trichuris trichiura (9.2%), and hookworms (3.1%). Of the total, 7.[illegible]% PSAC were infected with two STH species. Most of the positive cases with STH w[illegible] showed low infection intensities, and 15.1% ascariasis cases were revealed the moderate infection intensities. It is found that 68.7% of PSAC w[illegible] treated with albendazole. This study also revealed households level data as, 39.4% used water from hand-dug well, 52.5% of [illegible]d to [illegible]ve ≥ 30 minutes to collect water, 77.5% did not treat water, and 48.9% not [illegible] hand washing facility.<br><br>Conclusions<br>This study showed that a significant proportion of pre-school age children are suffering from STH infections at the study area despite provision of preventive chemotherapy distribution. Also, gaps in the intervention against STH were highlighted. Thus, a call for action is required to control and eliminate STH among PSAC in Ethiopia.
**Order of Authors:**	Mekuria Asnakew Asfaw
	Tigist Gezmu
	Teklu Wegayehu
	Alemayehu Bekele
	Zeleke Hailemariam
	Nebiyu Masresha

Powered by Editorial Manager® and ProduXion Manager® from Aries Systems Corporation

	Teshome Gebre
**Additional Information:**	
**Question**	**Response**
**Financial Disclosure**<br><br>Enter a financial disclosure statement that describes the sources of funding for the work included in this submission. Review the submission guidelines for detailed requirements. View published research articles from *PLOS ONE* for specific examples.<br><br>This statement is required for submission and **will appear in the published article** if the submission is accepted. Please make sure it is accurate.<br><br>**Unfunded studies**<br>Enter: *The author(s) received no specific funding for this work.*<br><br>**Funded studies**<br>Enter a statement with the following details:<br>• Initials of the authors who received each award<br>• Grant numbers awarded to each author<br>• The full name of each funder<br>• URL of each funder website<br>• Did the sponsors or funders play any role in the study design, data collection and analysis, decision to publish, or preparation of the manuscript?<br>• **NO** - Include this sentence at the end of your statement: *The funders had no role in study design, data collection and analysis, decision to publish, or preparation of the manuscript.*<br>• **YES** - Specify the role(s) played.<br><br>* typeset	This study is made possible by the generous research grant support of collaborative research and training center for NTDs, Arba Minch University, Ethiopia.
**Competing Interests**<br><br>Use the instructions below to enter a competing interest statement for this submission. On behalf of all authors, disclose any competing interests that could be perceived to bias this work—acknowledging all financial support and any other relevant financial or non-financial competing interests.	The authors have declared that no competing interests exist.

This statement **will appear in the published article** if the submission is accepted. Please make sure it is accurate. View published research articles from *PLOS ONE* for specific examples.<br><br>**NO authors have competing interests**<br><br>Enter: *The authors have declared that no competing interests exist.*<br><br>**Authors with competing interests**<br><br>Enter competing interest details beginning with this statement:<br><br>*I have read the journal's policy and the authors of this manuscript have the following competing interests: [insert competing interests here]*<br><br>* typeset	
**Ethics Statement**<br><br>Enter an ethics statement for this submission. This statement is required if the study involved:<br><br>• Human participants<br>• Human specimens or tissue<br>• Vertebrate animals or cephalopods<br>• Vertebrate embryos or tissues<br>• Field research<br><br>Write "N/A" if the submission does not require an ethics statement.<br><br>General guidance is provided below. Consult the submission guidelines for detailed instructions. **Make sure that all information entered here is included in the Methods section of the manuscript.**	Ethical approval with reference number CMHS/11222/111 was obtained from Institutional and Research Ethics review board of Arba Minch University, College of Medicine and Health Sciences, Ethiopia. Oral and written consent were obtained from district administrators and heads of the household before survey was conducted. Children tested positive for one or more STH were treated with albendazole or mebendazole by health workers.

Powered by Editorial Manager® and ProduXion Manager® from Aries Systems Corporation

**Format for specific study types**<br><br>**Human Subject Research (involving human participants and/or tissue)**<br>• Give the name of the institutional review board or ethics committee that approved the study<br>• Include the approval number and/or a statement indicating approval of this research<br>• Indicate the form of consent obtained (written/oral) or the reason that consent was not obtained (e.g. the data were analyzed anonymously)<br><br>**Animal Research (involving vertebrate animals, embryos or tissues)**<br>• Provide the name of the Institutional Animal Care and Use Committee (IACUC) or other relevant ethics board that reviewed the study protocol, and indicate whether they approved this research or granted a formal waiver of ethical approval<br>• Include an approval number if one was obtained<br>• If the study involved *non-human primates*, add *additional details* about animal welfare and steps taken to ameliorate suffering<br>• If anesthesia, euthanasia, or any kind of animal sacrifice is part of the study, include briefly which substances and/or methods were applied<br><br>**Field Research**<br><br>Include the following details if this study involves the collection of plant, animal, or other materials from a natural setting:<br>• Field permit number<br>• Name of the institution or relevant body that granted permission	
**Data Availability**<br><br>Authors are required to make all data underlying the findings described fully available, without restriction, and from the time of publication. PLOS allows rare exceptions to address legal and ethical concerns. See the PLOS Data Policy and FAQ for detailed information.	Yes - all data are fully available without restriction

Powered by Editorial Manager® and ProduXion Manager® from Aries Systems Corporation

A Data Availability Statement describing where the data can be found is required at submission. Your answers to this question constitute the Data Availability Statement and **will be published in the article**, if accepted.<br><br>**Important:** Stating 'data available on request from the author' is not sufficient. If your data are only available upon request, select 'No' for the first question and explain your exceptional situation in the text box.<br><br>Do the authors confirm that all data underlying the findings described in their manuscript are fully available without restriction?	
**Describe where the data may be found in full sentences. If you are copying our sample text, replace any instances of XXX with the appropriate details.**<br><br>• If the data are **held or will be held in a public repository**, include URLs, accession numbers or DOIs. If this information will only be available after acceptance, indicate this by ticking the box below. For example: *All XXX files are available from the XXX database (accession number(s) XXX, XXX.*).<br>• If the data are all contained **within the manuscript and/or Supporting Information files**, enter the following: *All relevant data are within the manuscript and its Supporting Information files.*<br>• If neither of these applies but you are able to provide **details of access elsewhere**, with or without limitations, please do so. For example:<br><br>*Data cannot be shared publicly because of [XXX]. Data are available from the XXX Institutional Data Access / Ethics Committee (contact via XXX) for researchers who meet the criteria for access to confidential data.*<br><br>*The data underlying the results presented in the study are available from (include the name of the third party*	All relevant data are within the manuscript.

and contact information or URL).<br>• This text is appropriate if the data are owned by a third party and authors do not have permission to share the data.<br><br>* typeset	
Additional data availability information:	

Powered by Editorial Manager® and ProduXion Manager® from Aries Systems Corporation

Manuscript

Click here to access/download;Manuscript;Mekuria et al_Plos_Article_final_revised_August 12.docx

Soil-transmitted helminth infections among pre-school aged children in Gamo Gofa zone, Southern Ethiopia: Prevalence, intensity and intervention status

Mekuria Asnakew Asfaw[1*], Tigist Gezmu[1], Teklu Wegayehu[2], Alemayehu Bekele[1], Zeleke Hailemariam[3], Nebiyu Masresha[4], Teshome Gebre[5]

[1]Collaborative Research and Training Centre for NTDs, Arba Minch University, Ethiopia

[2]Department of Biology, College of Natural Sciences, Arba Minch University, Ethiopia

[3]School of Public health, College of Medicine and Health Sciences, Arba Minch University, Ethiopia

[4] Ethiopian Public Health Institute, Addis Ababa, Ethiopia

[5] The Task Force for Global Health, International Trachoma Initiative, Addis Ababa, Ethiopia

[*]**Corresponding author**

E-mail : maksambaramr23@gmail.com (MA)

Abstract

Background

Soil-transmitted helminths (STH), i.e., *Ascaris lumbricoides*, *Trichuris trichiura* and hookworms are among the most prevalent neglected tropical diseases (NTDs) in Ethiopia. Although pre-school a[illegible]children pa[illegible]gh morbidity toll associated with STH infections, evidence on prevalence, intensity and intervention status is lacking in Ethiopia. This study, therefore, aimed to address these gaps to inform decision made on STH.

Methods

We did a community-based cross-sectional study in five districts of Gamo Gofa zone, southern Ethiopia; in January 2019. Data were collected using pre-tested questionnaire, and the Kato-Katz technique was used to diagnose parasites egg in stool. Then, the collected data were edited and entered into EpiData 4.4.2, and exported to SPSS software (IBM, version 25) for analysis.

Results

A total of 2462 PSAC participated in this study. Overall, the prevalence of STH was 23.5% (578/2462) (95% confidence interval (CI) = 21.8–25.2%) with highest prevalence (33.8%) in Chencha district. As*caris lumbricoides* was the most prevalent (18.6%), followed by *Trichuris trichiura* (9.2%), and hookworms (3.1%). Of the total, 7.4% PSAC were infected with two STH species. Most of the positive cases with STH were showed low infection intensities while 15.1% *ascariasis* cases were revealed the moderate infection intensities. It is found that 68.7% of PSAC were treated with albendazole. Also, household’s level data showed that 39.4% used water from hand-dug well, and 48.9% not had hand washing facility. In addition, almost 93% mothers or guardians achieved less than the mean knowledge and practice score (≤5) on STH prevention.

Conclusions

This study showed that significant proportion of pre-school age children are suffering from STH infections despite provision of mass drug administration at the study area. Also, gaps in the intervention against STH were highlighted. Thus, a call for action is demanding to control and eliminate STH among PSAC in Ethiopia.

Introduction

Soil-transmitted helminth (STH), i.e., *Ascaris lumbricoides, Trichuris trichiura* and hookworms infections are among the most common neglected tropical diseases (NTDs) [1]. It is prevalent mainly in tropical and subtropical areas where water supply, hygiene and sanitation infrastructure are inadequate [2-4] . Moderate and heavy infections intensity of STH are associated with chronic harmful effects on vitamin A and iron status, physical, intellectual, and cognitive development in pre-school age children (PSAC), these morbidities not only take a huge toll on the health of children, but has also been shown to affect economic development of a nation [5, 6] .

Globally, over 2 billion people are affected with STH where ascariasis accounts for almost 1.2 billion infections while trichiurasis and hookworms (*Ancylostoma duodenale* and *Necator americanus*) responsible for over 800 million and 740 million infections, respectively [1, 7, 8] . The global burden of STH infections is estimated at between 5 and 39 million disability-adjusted life years (DALYs), and in 2010, 5.18 DALYs were estimated as associated with STH infections [9, 10]. The greater burden of STH infections is found in the tropical countries including tropical South America, China, Southeast Asia, and Sub-Saharan Africa [5]. According to the WHO estimate 42 countries in Africa are endemic for STH with 284 million cases occurring in both school age and pre-school age children. These children require periodic administration of preventive chemotherapy [1, 11].

In Ethiopia, the number of people living in STH endemic areas is estimated at 81 million, of which pre-school age children accoun[illegible]1 million [12]. The greatest numbers of intestinal worms harbored in children resulting in diarrhea, loss of appetite, weight loss, growth retardation, malnutrition, anemia and cognitive defects [6, 13-16].

In 2012, the World health organization (WHO) endorsed road map to combat NTDs by 2020, and substantial progress has been made in terms of controlling STH associated morbidity [3]. The WHO goal is to reduce the prevalence of moderate and heavy infections with soil transmitted helminths among preschool and school aged children to below 1%, to be no longer considered public health problems by 2020 [17].

In line [illegible]HO's goal, Ethiopia has also set a similar goal to achieve by the same year [12]. To achieve these goals, in areas where prevalence of any soil-transmitted infection is 20% or higher, periodic mass administration of preventive chemotherapy (deworming) using annual or biannual single-dose albendazole or mebendazole is recommended by WHO for all pre-school and school age children (10).

As part of the global action towards Universal Health Coverage (UHC), ending NTDs is prioritized by 2030 in the Sustainable Development Goal (SDG) agenda under target 3.3 [3]. Moreover, working on NTDs helps the vision of universal health coverage, which means that all individuals and communities access the health services they need without suffering from financial suffering [18].

Since 2008 preventive chemotherapy (PC) against STH in PSAC has been implemented alongside Vitamin A distribution in the study area as well as at national level through community based drug distribution platforms [12]. In 2013, the first national master plan for NTDs was launched, and then the government of Ethiopia has been collaborating with the WHO and other partners for mapping all endemic districts to address SAC through the school based mass drug administration [19]. This implies that PC was started before ten years

to combat STH in PSAC at study area. However, the impact of deworming on STH infection status among PSAC is not monitored and evaluated, and evidence is lacking at national level on prevalence, intensity and intervention status of STH infections among PSAC. Therefore, the present study aimed to determine prevalence, intensity, and intervention status of STH infections among preschool children in Gamo Gofa zone and to inform decision-making. .

Methods

Study area and period

This study was conducted in the former Gamo Gofa zone, southern Ethiopia; in January 2019. The zone is found in Southern Nations, Nationalities, and Peoples' Regional State (SNNPR) and it had 15 districts and two city administrations (CAs). All districts and CAs are endemic for STH, 15 had moderate prevalence and two had low prevalence. According to the 2007 estimate of Central Statistical Agency of Ethiopia, a total of 2,043,668 people live in the zone, of which 1,013,533 are males and 1,030,135 are females [20]. Arba Minch is the capital city of the zone, which is located at 437 km away from Addis Ababa, capital city of Ethiopia.

Study design and populations

Community-based cross-sectional study was conducted. The study population was all PSAC in the selected STH endemic *Kebeles* (localities). Since there is no consistent definition of PSAC in the existing literatures, we have used age as any child between 1 and 5 years to include participants. PSAC who were unable to give stool samples at the time of collection were excluded from the study. Additionally, PSAC were excluded in case when they were seriously ill or parents were unable to provide their information.

Determining sample size and sampling technique

The sample size was determined using single population proportion formula,

$$\mathbf{n} = \frac{\left(Z_{\left(\frac{\alpha}{2}\right)}\right)^2 P(1-P)}{d^2},$$

assuming p, **25.7%** (proportion of PSAC infected with STH) [21]; **Z,** 1.96 at significant level of alpha **(α)** of **0.05**, and desired degree of precision (d) of **3 %**, and design effect=**3**. The computed sample size was **2434** and by adding **10%** non-response rate, the total computed sample size was **2678.**

Multi-stage cluster sampling technique was employed in order to select study participants (Figure 1). First, districts and CAs STH endemicity status was identified based on results of previous STH mapping survey conducted at national level (where 2 had low and 15 had moderate prevalence level [22]. We excluded 2 districts with low endemicity status since they were not eligible for preventive chemotherapy. Second, 5 districts and 12 *kebeles* were selected from 15 districts by simple random sampling technique (SRS). Third, list of eligible households in each *kebeles* which had children between 1 and 5 years of age were identified by health extension workers (HEWs). Finally, children from each *kebeles* were selected by taking probability proportionate to sample size into account.

Figure 1. Sampling profile

Study variables and data collection

Variables included in this study were STH infection status (positive or negative), intensity of infections, socio-demographic and economic characteristics of parent and guardians, social determinants of health, wealth status of household and child related variables and implementation of STH control strategies. Data on these socio-demographic and other variables were collected through face-to-face interviews using pre-tested questionnaire with heads of households or mothers or guardians. Stool specimens were examined using the WHO recommended Kato-Katz kit under microscopy [23].

Stool collection and processing

Fresh stool specimens were collected using clean, leak proof and screw cup container, and then the collected stool samples were transported in an icebox to nearby health facility for

examination. The stool samples were processed within two hours of receipt or saved in an icebox where travel time exceeded two hours. Samples were examined in the local health center by Kato-Katz technique to determine the prevalence and intensity of STH.

Quality control

Data quality was monitored through standard operational procedure, recruiting competent data collectors, pre-testing tools, training data collectors and supervisors, daily checking consistency and accuracy of collected data. The quality of data collection was closely monitored by supervisors. Stools were examined by qualified laboratory technicians. Duplicate slides were prepared per stool sample in order to ensure reliability. For the purpose of bench aid, pictures of parasites egg were displayed on wall in front of microscopy examination for the purpose of internal quality control.

Statistical analysis and measurements

First, data were edited and entered into EpiData 4.4.2, and then exported to SPSS software (IBM, version 25) for analysis. A difference in prevalence of STH between or among categories of variables was analyzed using chi-square test (X^2). Summary statistics were computed, and data were presented with tables and graphs. Children between age 1 and 5 years were defined as PSAC [23].

Wealth analysis was performed as, initially, reliability test was performed using the economic variables involved in measuring the wealth of the households. The variables which were used to compute the alpha value were entered into the principal component analysis. At the end of the principal component analysis, the wealth index was obtained as a continuous scale of relative wealth. Then, quintiles of the wealth index were created. Knowledge and practice on STH transmission and prevention was measured using 11 questions and score was computed by counting value within a case.

We calculated the prevalence by dividing the number of STH positive PSAC by the total number of participants. Intensity of STH infection is the number of helminths (worms) infecting an individual; for each parasite species it was analyzed as light, moderate and heavy infections based on number of eggs per gram of stool (EPG), and it was classified according to the WHO guidelines [24] as follow (Table 1).

Table 1. Criteria for classifying STH infection intensity for each species.

STH species	Intensity of infection (EPG)		
	**Light**	**Moderate**	**Heavy**
A. lumbricoides	(1–4999)	(5000–49999)	(≥50000)
T. trichiura	(1–999)	(1000–9999)	(≥10000)
**Hookworms**	(1–1999)	(2000–3999)	(≥4000)

According to WHO STH endemicity mapping classifications, there are three categories in line with implementation of mass drug administration (MDA): i) high transmission (where prevalence is > 50%), ii) moderate transmission (where prevalence is between 20%- 50%), and iii) low transmission (where prevalence is < 20%) [23, 25].

Ethics statement

Ethical approval with reference number CMHS/11222/111 was obtained from Institutional and Research Ethics review board of Arba Minch University, College of Medicine and Health Sciences, Ethiopia. Oral and written consent were obtained from district administrators and heads of the household before survey was conducted. Children tested positive for one or more STH were treated with albendazole or mebendazole by health workers.

Results

Socio-demographic and economic characteristics

Details on socio-demographic and economic characteristics are presented in Table 2. A total of 2462 PSAC participated in this study. Of the total, 246 (10%) were under 2 years of age,

and slightly more males participated than females (52% versus 48%). More than half of HHs (57.5%) did not attend any formal education (Table 2).

Table 2. Socio-demographic characteristics of PSAC and HHs, 2019 (N=2462).

**Variable**	**Category**	**Frequency**	**Percent (%)**
**Child sex**	Male	1281	52
	Female	1181	48
**Child age (years)**	Less than 2yrs	246	10
	2-5yrs	2216	90
**Age of HH (years)**	Less than 20	22	0.89
	20 -29	404	16.41
	30-39	1284	52.15
	40-49	647	26.28
	55-59	59	2.4
	60 and above	46	1.87
**Sex of HH**	Male	2174	88.3
	Female	288	11.7
**Educational status**	No formal education	1416	57.51
	Elementary	508	20.63
	Secondary	279	11.33
	Diploma and above	259	10.52
**Occupation**	Farming	1606	65.23
	Employed	312	12.67
	Merchant	303	12.31
	Unemployed	78	3.17
	Others*	163	6.62
**Family size**	Less than 4	288	11.7
	4-6	1503	61.05
	7 and more	671	27.25
**Residence**	Urban	628	25.5
	Rural	1834	74.5
**Wealth quintile**	Wealthiest	488	19.82
	Wealthy	498	20.22
	Middle income	482	19.58
	Poor	498	20.23
	Poorest	496	20.15
**Water source**	Pipe	1128	45.82
	Well	293	11.9
	Public bono	971	39.44
	Other**	70	2.84

*=Daily laborer; **=river, well and spring

Prevalence of STH infections

Of the total surveyed children, 23.5% (578/2462) (95% confidence interval (CI) = 21.8–25.2%) had at least one type of STH infection. Ascariasis was the most prevalent (18.6%), followed by trichiurasis (9.2%) and hookworms (3.1%). Mixed STH infections (*Ascaris*

lumbricoides and *Trichuris trichiura*) were found in 7.4% of PSAC. The highest prevalence (33.8 %) of any one of STH infection was observed in Chencha district, as contrasted to the lowest prevalence (11%) was found in Demba Gofa district. In Deremalo district, considerable amount of hookworm infections (10%) were revealed (Figure 2).

Figure 2.Prevalence of any STH infection dis-aggregated by districts among PSAC, Gamo Gofa zone, Southern Ethiopia, 2019 (N=2462).

The prevalence of STH infections across the age-group (1 – 5 years) was slightly higher at ≤2 years while comparing across the age-group (1-5 years). On the other hand, prevalence of any STH infections among females (24.3%) was a little higher than the male's (22.7%), and a higher prevalence of STH infections (24.6%) in rural area was noticed than urban (20.1%). Children who did not receive PC in the last one year had slightly higher prevalence of STH infections compared to those who received PC (23.9% versus 18.7%), (Table 3).

Table 3. Prevalence of STH infections among PSAC dis-aggregated by different factors in Gamo Gofa zone, 2019 (N=2462).

Variable	Category	STH Infection status		X2-test	P-value
		Negative, n (%)	Positive, n (%)		
**Child sex**	Male	990 (77.3)	291 (22.7)	0.354	0.366
	Female	894 (75.7)	287 (24.3)		
**Child age group (years)**	Less than 2yrs	187 (76)	59 (24)	0.039	0.843
	2-5yrs	1697 (76.6)	519 (23.4)		
**Place of residence**	Urban	502 (79.9)	126 (20.1)	**5.467**	**0.019**
	Rural	1382 (75.4)	452 (24.6)		
**Child age (years)**	1	187 (76)	59 (24)	0.914	0.923
	2	414 (75.1)	137 (24.9)		
	3	563 (77)	168 (23)		
	4	522 (77.2)	154 (22.8)		
	5	198 (76.7)	60 (23.3)		
**Dew[illegible]ed in the last [illegible] year**	No	608 (78.9)	163 (21.1)	3.408	0.065
	Yes	1276 (75.5)	415 (24.5)		
**Child soil eating habit**	No	1518 (76.9)	457 (23.1)	0.371	0.542
	Yes	261 (78.4)	72 (21.6)		
**Family size**	<4	220 (76.4)	68 (23.6)	2.219	0.330
	4-6	1164 (77.4)	339 (22.6)		
	7 and above	500 (74.5)	171 (25.5)		
**Mothers' (guardians') educational status**	Can't read and write	774 (76.9)	233 (23.1)	5.432	0.246
	Can read and write	291 (73.9)	103 (26.1)		
	Elementary	486 (76.1)	153 (23.9)		
	Secondary	225 (76.8)	68 (23.2)		
	Diploma and above	108 (83.7)	21 (16.3)		
**Mothers' (guardians') occupation**	Farming	974 (74.6)	331 (25.4)	**16.288**	**0.003**
	Employed (Gov.)	166 (81.8)	37 (18.2)		
	Merchant	348 (73.7)	124 (26.3)		
	Unemployed	334 (82.3)	72 (17.7)		
	Others*	62 (81.6)	14 (18.4)		
**Wealth quintile**	Highest	379 (77.7)	109 (22.3)	1.372	0.712
	Fourth	379 (76.1)	119 (23.9)		
	Middle income	374 (77.6)	108 (22.4)		
	Second	369 (74.1)	129 (25.9)		
	Lowest	383 (77.2)	113 (22.8)		
**Water source**	Pipe	891 (79)	237 (21)	**8.289**	**0.04**
	Well	223 (76.1)	70 (23.9)		
	Public bono	721 (74.3)	250 (25.7)		
	Other**	49 (70)	21 (30)		

*****=Daily laborer and housewife ******=River, well and spring

STH infections intensity

In majority of STH infections (85%), low infection intensity was found as a result of hookworms and *Trichuris trichiura* while 15% of Ascariasis had moderate infection intensity (Table 4). All of the moderate infections were from Chencha and Bonke districts.

Table 4. STH infection Intensity among PSAC in Gamo Gofa zone, southern Ethiopia, 2019 (N=2462).

Type of STH infection	Mean (EPG)	Infection intensity		
		Light	Moderate	Total infected PSAC
**Ascariasis**	2152	388 (84.90)	69 (15.1)	457
**Hookworms**	154	76 (100)	0	76
**Trichiurasis**	135	226 (100)	0	226

Intervention status against STH infections

I. PC treatment coverage against STH

The overall self-reported treatment coverage with albendazole (ALB) against STH among PSAC was 68.7% (1691/2462) in the last one year before the survey.

II. Knowledge and practice (KP) of mothers or guardians related to STH transmission

Table 5 presents the details on KP on STH transmission among mothers or guardians. Of the total surveyed mothers or guardians, almost 93% (2283/2462) achieved less than the mean KP score (≤5) on prevention of STH, 42.7% (1052/2462) did not wash hand after defecation, and 77.7% **(**1913/2462) got information on STH from health extension workers (HEWs) (Table 5**).**

Table 5. Knowledge and practice (KP) of mothers or guardians related to STH transmission, 2019 (N=2462).

Variables	Category	Frequency	%
**Knew about STH**	Yes	2208	89.7
	No	254	10.3
**Know at least one STH transmission (n=2208)**	Yes	2153	97.5
	No	55	2.5
**Know at least one STH prevention way (n=2208)**	Yes	2135	96.7
	No	73	13.5
**KP score on STH transmission**	≤5	2262	91.9
	>5	200	8.1
**KP score on STH prevention**	≤5	2283	92.7
	>5	179	7.3
**Source of information about STH**	Health facility	271	11
	HEWs	1913	77.7
	Radio or TV	24	1
	None	254	10.3
**Wash hand after latrine**	Yes	1410	57.3
	No	1052	42.7
**Wash hand before meal**	Yes	2347	95.3
	No	2462	4.7
**Wash hand after cleaning child**	Yes	2229	90.5
	No	233	9.5
**Wash hand before cooking**	Yes	2069	84
	No	393	16
**Wash fruit before eating**	Yes	1841	74.8
	No	621	25.2
**Wash hand after work**	Yes	2253	91.5
	No	209	8.5
**Where do you dispose child faeces?**	Within home compound	194	7.9
	Toilet	2002	81.3
	Garbage	266	10.8

III. Water, Sanitation and Hygiene (WASH)

Table 6 presents the details on WASH characteristics at HHs level. This study also revealed households data as, 39.4% used water from hand-dug well, 52.5% of need to move more than 30 minutes to collect water, 77.5% did not use treat water, and 48.9% did not own hand washing facility (Table 6).

Table 6. WASH characteristics of households in Gamo Gofa zone, 2019.

Variables	Category	Frequency	%
**Source of water**	Pipe	1128	45.82
	Well	293	11.9
	Public bono	971	39.44
	Other*	70	2.84
**Distance from water source**	< 30min	1170	47.5
	≥ 30min	1292	52.5
**Adequate water**	No	536	21.8
	Yes	1926	78.2
**Ever treat water**	No	1909	77.5
	Yes	553	22.5
**Have latrine**	Yes	2397	97.4
	No	65	2.6
**Type of latrine (n=2397)**	Pit	2360	98.5
	Improved pit latrine	37	1.5
**Latrine clean**	No	1766	73.67
	Yes	631	26.32
**Hand washing facility (functional)**	No	1225	51.1
	Yes	1172	48.9
**Soap or ash available (n=1172)**	Yes	298	25.4
	No	874	74.6
**Reason fo[illegible]t latrine available (n=65)**	No space	6	9.2
	No money	10	15.4
	No skill	9	13.8
	Did not know importance	40	61.5
**If no latrine, where do you defecate (n=65**	Open field	56	86.2
	Public	9	13.8

*=river, well and spring

Discussion

This study provides operational evidence on prevalence, intensity and intervention status of soil-transmitted helminth infections (STH) among PSAC. Our study showed that a significant proportion of PSAC are suffering from STH infections despite provision of mass drug administration at the study area. Also, gaps in the intervention against STH to control and eliminate STH were highlighted.

In this study, the overall prevalence of STH infections with at least one STH parasite was 23.5%, would be classified into the moderate transmission category (where prevalence is between 20% and 50%), and qualifies the requirement of annual STH mass drug administration [17]. Ascariasis was the most prevalent infection (18.6%), followed by

trichiurasis (9.2%) and hookworms (3.1%). Most of the positive cases with STH were showed low infection intensities, and 15.1% cases of ascariasis were revealed the moderate infection intensities. Despite initiation of preventive chemotherapy about 10 years ago, the burden of STH did not show significant reduction probably due to weak implementation of control strategies, such as social behavioral change communication (SBCC), inadequate mass drug administration coverage and WASH, as highlighted in the findings of this study.

The overall prevalence of STH infections observed in this study is comparable with studies conducted in another parts of Ethiopia (Butajira and Wonji) and West China [26-28], but slightly lower than the prevalence reported in Dembiya, northwest Ethiopia [21]. In addition, the prevalence in our study is significantly lower than the findings of other studies conducted in another part of Ethiopia and in Nigeria, Cameroon, Ecuador, Uganda, Kenya and Honduras [29-36]. These differences observed from our study could be due to variation in socio-cultural, social determinants, behavioral characteristics and implementation of prevention and control measures.

In our study, Ascariasis was identified as the commonest species of STH, and this findings supports studies done in another part of Ethiopia, Nigeria and China [21, 26, 28, 29]. On the contrary, some other studies conducted in Ethiopia, Ecuador and Honduras showed high prevalence of Trichiurasis [32, 35, 37], and a study conducted in Uganda showed high prevalence of hookworm [33]. These differences might be related to variation in environmental factors such as climate, rainfall, topography, surface temperature, altitude, and soil type which have a great impact on the distribution of STH [38]. Moreover, in this study we found significant amounts of mixed infections, 7.4% of PSAC were infected with two STH species (*Ascaris lumbricodes and Trichuris trichiura*); this finding is in line with a study conducted in another areas of Ethiopia and Nigeria [26, 27, 29].

In this study, slightly higher prevalence at age $\leq$2 years was observed while comparing across the age-group (1-5 years); the possible explanation related to this difference is due to the fact that current mass drug administration among PSAC often do not include age $\leq$2 years. On the contrary, other studies revealed numerical increase in prevalence of STH as age increase [26, 29]. In addition, in this study, prevalence of any STH infection among females (24.3%) was a little higher than the male's prevalence (22.7%), this result supports the findings of a study conducted in another part of Ethiopia [26]. These differences might be due to low access and uptake of preventive chemotherapy among females, as justified by data of our study.

In addition, significant proportion (15%) of moderate intensity Ascariasis was observed in our study, and this finding is higher than results of a study conducted in Butajira, Ethiopia and Honduras [26, 35]. The possible explanation related to this difference could be consistency and frequency of mass drug administration that may affect intensity of infections [23].

Furthermore, in our study, gaps in the intervention against STH among PSAC were highlighted. The treatment coverage of albendazole (ALB) in this survey against STH among PSAC was (68.7%); which is lower than the national coverage (71%) of Ethiopia and WHO's target (minimum of 75%) [10, 39]. Most importantly, study participants in this study were pre-school age children, who might not be reached out by the deworming program, especially those under 2 years old. On the other hand, obviously, school age children could have better chance to be reached by school-based deworming.

Although site-specific data are required in our case, the importance of WASH intervention to control and eliminate STH was reported in different studies [40-42]. However, in this study inadequate WASH infrastructures were observed at household level, where 39.4% were using water from well, 52.5% were walking more than 30 minutes to collect water, 77.5 % did not

treat water, and 48.9% of not had hand washing facility. Of the total surveyed mothers or guardians, almost 93% achieved less than the mean KP score (≤5) on prevention of STH, 42.7% did not wash hand after defecation, and 77.7% got information on STH from health extension workers (HEWs). The possible reason for these findings could be related to weak and inconsistent social behavioral change communication (SBCC) intervention.

The most outstanding strength of our study is that it is addressing an important national operational research priority which is focusing on parasitological monitoring and control strategies of STH among pre-school age children.

In this study, there are limitations that need to be taken into account. There might be underestimation of prevalence of STH due to the fact that (1) we collected single stool specimen, which could cause variation in egg excretion over different times (hours) within a day and across different days; (2) samples were collected from remote villages and there might be rapid desiccation of hookworm eggs in the stool samples, and (3) even though the Kato–Katz technique is sensitive in detecting moderate and high infection intensity (MHI), it has lower detection power and therefore lower positive predictive values in low-prevalence (and low-intensity) settings [28, 43].

Conclusions

Data from our study showed that substantive proportion of pre-school age children in the study area are suffering from significant burden of STH infections despite provision of preventive chemotherapy distribution. Also, gaps in the intervention against STH to control and eliminate STH were highlighted.

Thus, a call for action is demanding to improve interventions against STH among PSAC to achieve the national goal of STH control and elimination in Ethiopia. Further, operational research needs to be conducted in different settings to monitor and evaluate progress towards elimination and control of STH among PSAC.

Acknowledgements

Our sincere goes to study participants, data collectors, supervisors, zonal health office heads, district health office heads, NTDs focal persons in all selected districts of the study area and Ethiopian public health institute for their support in the implementation of the study.

References

1. Montresor A, Trouleau W, Mupfasoni D, Bangert M, Joseph SA, Mikhailov A, et al. Preventive chemotherapy to control soil-transmitted helminthiasis averted more than 500 000 DALYs in 2015. Transactions of the Royal Society of Tropical Medicine and Hygiene. 2017 Oct 1; 111(10):457-63.
2. Hotez PJ, Fenwick A, Savioli L, Molyneux DH. Rescuing the bottom billion through control of neglected tropical diseases. The Lancet. 2009 May 2; 373(9674):1570-5.
3. World Health Organization. Ending the neglect to attain the sustainable development goals: A road map for neglected tropical diseases 2021–2030. World Health Organization; 2020.
4. De Silva NR, Brooker S, Hotez PJ, Montresor A, Engels D, Savioli L. Soil-transmitted helminth infections: updating the global picture. Trends in parasitology. 2003 Dec 1; 19(12):547-51.
5. Bethony J, Brooker S, Albonico M, Geiger SM, Loukas A, Diemert D, et al. Soil-transmitted helminth infections: ascariasis, trichuriasis, and hookworm. The Lancet. 2006 May 6; 367(9521):1521-32.
6. Albonico M, Allen H, Chitsulo L, Engels D, Gabrielli AF, Savioli L. Controlling soil-transmitted helminthiasis in pre-school-age children through preventive chemotherapy. PLoS neglected tropical diseases. 2008 Mar; 2(3).
7. Schistosomiasis WH. soil-transmitted helminth infections–preliminary estimates of the number of children treated with albendazole or mebendazole. Wkly Epidemiol Rec. 2006 Apr 21; 81(16):145-64.
8. Pullan RL, Brooker SJ. The global limits and population at risk of soil-transmitted helminth infections in 2010. Parasites & vectors. 2012 Dec 1; 5(1):81.

9. Brooker S. Estimating the global distribution and disease burden of intestinal nematode infections: adding up the numbers–a review. International journal for parasitology. 2010 Aug 15; 40(10):1137-44.
10. World Health Organization. Guideline: preventive chemotherapy to control soil-transmitted helminth infections in at-risk population groups. World Health Organization; 2017.
11. World Health Organization. Intestinal worms: Soil-transmitted helminthes World Health Organization; 2011.
12. Federal Democratic Republic of Ethiopia Ministry of Health. Second Edition of Ethiopia National Master Plan for Neglected Tropical Diseases. Addis Ababa, Ethiopia. 2016.
13. Tchuenté LA. Control of schistosomiasis and soil-transmitted helminthiasis in sub-Saharan Africa: challenges and prospects. Current topics in tropical medicine. 2012 Mar 16:359-76.
14. Stephenson LS, Latham MC, Ottesen EA. Malnutrition and parasitic helminth infections. Parasitology. 2000 Oct; 121(S1):S23-38.
15. Soeripto N. Reinfection and infection rates of soil-transmitted-helminths in Kemiri Sewu, Yogyakarta, Indonesia. Southeast Asian J Trop Med Public Health. 1991 Jun; 22(2):216-21.
16. Schistosomiasis WH. soil-transmitted helminth infections–preliminary estimates of the number of children treated with albendazole or mebendazole. Wkly Epidemiol Rec. 2006 Apr 21; 81(16):145-64.
17. World Health Organization. Soil-transmitted helminthiases: eliminating as public health problem soil-transmitted helminthiases in children: progress report 2001-2010 and strategic plan 2011-2020.
18. World Health Organization. Universal health coverage. WHO fact sheet. Geneva. World Health Organization; 2019.
19. Federal Democratic Republic of Ethiopia Ministry of Health. Ethiopian National Master Plan for Neglected Tropical Diseases (2013-2015). Addis Ababa, Ethiopia; 2013.
20. Central statistical agency (2013) Population Projection for Ethiopia 2007-2037. Addis Ababa, Ethiopia.

21. Gizaw Z, Adane T, Azanaw J, Addisu A, Haile D. Childhood intestinal parasitic infection and sanitation predictors in rural Dembiya, northwest Ethiopia. Environmental health and preventive medicine. 2018 Dec; 23(1):26.

22. Leta GT, Mekete K, Wuletaw Y, Gebretsadik A, Sime H, Mekasha S, et al. National mapping of soil-transmitted helminth and schistosome infections in Ethiopia.

23. World Health Organization. Preventive chemotherapy in human helminthiasis. Coordinated use of anthelminthic drugs in control interventions: a manual for health professionals and programme managers. World Health Organization; 2006.

24. World Health Organization. Prevention and control of schistosomiasis and soil-transmitted helminthiasis: report of a WHO expert committee.

25. Helminthiases WS. Eliminating soil-transmitted helminthiases as a public health problem in children: progress report 2001–2010 and strategic plan 2011–2020. France: World Health Organization. 2012:19-29.

26. Shumbej T, Belay T, Mekonnen Z, Tefera T, Zemene E. Soil-transmitted helminths and associated factors among pre-school children in Butajira Town, South-Central Ethiopia: a community-based cross-sectional study. PloS one. 2015; 10(8).

27. Degarege A, Erko B. Prevalence of intestinal parasitic infections among children under five years of age with emphasis on Schistosoma mansoni in Wonji Shoa Sugar Estate, Ethiopia. PloS one. 2014; 9(10).

28. Wang X, Zhang L, Luo R, Wang G, Chen Y, Medina A, et al. Soil-transmitted helminth infections and correlated risk factors in preschool and school-aged children in rural southwest China. PLoS One. 2012; 7(9).

29. Sowemimo OA, Asaolu SO. Current status of soil-transmitted helminthiases among pre-school and school-aged children from Ile-Ife, Osun State, Nigeria. Journal of helminthology. 2011 Sep; 85(3):234-8.

30. Nkengazong L, Njiokou F, Wanji S, Teukeng F, Enyong P, Asonganyi T. Prevalence of soil transmitted helminths and impact of Albendazole on parasitic indices in Kotto Barombi and

Marumba II villages (South-West Cameroon). African Journal of Environmental Science and Technology. 2010; 4(3).

31. Dana D, Mekonnen Z, Emana D, Ayana M, Getachew M, Workneh N, et al. Prevalence and intensity of soil-transmitted helminth infections among pre-school age children in 12 kindergartens in Jimma Town, southwest Ethiopia. Transactions of the Royal Society of Tropical Medicine and Hygiene. 2015 Mar 1; 109(3):225-7.
32. Moncayo AL, Lovato R, Cooper PJ. Soil-transmitted helminth infections and nutritional status in Ecuador: findings from a national survey and implications for control strategies. BMJ open. 2018 Apr 1; 8(4):e021319.
33. Ojja S, Kisaka S, Ediau M, Tuhebwe D, Kisakye AN, Halage AA, et al. Prevalence, intensity and factors associated with soil-transmitted helminths infections among preschool-age children in Hoima district, rural western Uganda. BMC infectious diseases. 2018 Dec 1; 18(1):408.
34. Davis SM, Worrell CM, Wiegand RE, Odero KO, Suchdev PS, Ruth LJ, et al. Soil-transmitted helminths in pre-school-aged and school-aged children in an urban slum: a cross-sectional study of prevalence, distribution, and associated exposures. The American jo[illegible]l of tropical medicine and hygiene. 2014 Nov 5; 91(5):1002-10.
35. Torres RE, Garcia DN, Sandoval GA, Santana AH, Singh P, Bucheli ST, et al. Prevalence and intensity of soil-transmitted helminthiasis, prevalence of malaria and nutritional status of school going children in Honduras. PLoS neglected tropical diseases. 2014 Oct; 8(10).
36. Tekalign E, Bajiro M, Ayana M, Tiruneh A, Belay T. Prevalence and Intensity of Soil-Transmitted Helminth Infection among Rural Community of Southwest Ethiopia: A Community-Based Study. BioMed Research International. 2019; 2019.
37. Nyantekyi LA, Legesse M, Belay M, Tadesse K, Manaye K, Macias C, et al. Intestinal parasitic infections among under-five children and maternal awareness about the infections in Shesha Kekele, Wondo Genet, Southern Ethiopia. Ethiopian Journal of Health Development. 2010; 24(3).

38. Hotez PJ, Brindley PJ, Bethony JM, King CH, Pearce EJ, Jacobson J. Helminth infections: the great neglected tropical diseases. The Journal of clinical investigation. 2008 Apr 1; 118(4):1311-21.

39. Asfaw M, Zerdo Z, Churko C, Seife F, Yihune M, Chisha Y, et al. Preventive chemotherapy coverage against Soil-transmitted helminthiases among school age children in vertical versus integrated treatment approaches: Implications from coverage validation survey in Ethiopia. medRxiv. 2020 Jan 1.

40. Freeman MC, Ogden S, Jacobson J, Abbott D, Addiss DG, Amnie AG, et al. Integration of water, sanitation, and hygiene for the prevention and control of neglected tropical diseases: a rationale for inter-sectoral collaboration. PLoS neglected tropical diseases. 2013 Sep; 7(9).

41. Ziegelbauer K, Speich B, Mäusezahl D, Bos R, Keiser J, Utzinger J. Effect of sanitation on soil-transmitted helminth infection: systematic review and meta-analysis. PLoS Med. 2012 Jan 24; 9(1):e1001162.

42. Becker SL, Liwanag HJ, Snyder JS, Akogun O, Belizario Jr V, Freeman MC, Gyorkos TW, Imtiaz R, Keiser J, Krolewiecki A, Levecke B. Toward the 2020 goal of soil-transmitted helminthiasis control and elimination. PLoS neglected tropical diseases. 2018 Aug; 12(8).

43. Freeman MC, Akogun O, Belizario Jr V, Brooker SJ, Gyorkos TW, Imtiaz R, et al. Challenges and opportunities for control and elimination of soil-transmitted helminth infection beyond 2020. PLoS neglected tropical diseases. 2019 Apr; 13(4).

Figure

Click here to access/download;Figure;Fig 1.tif

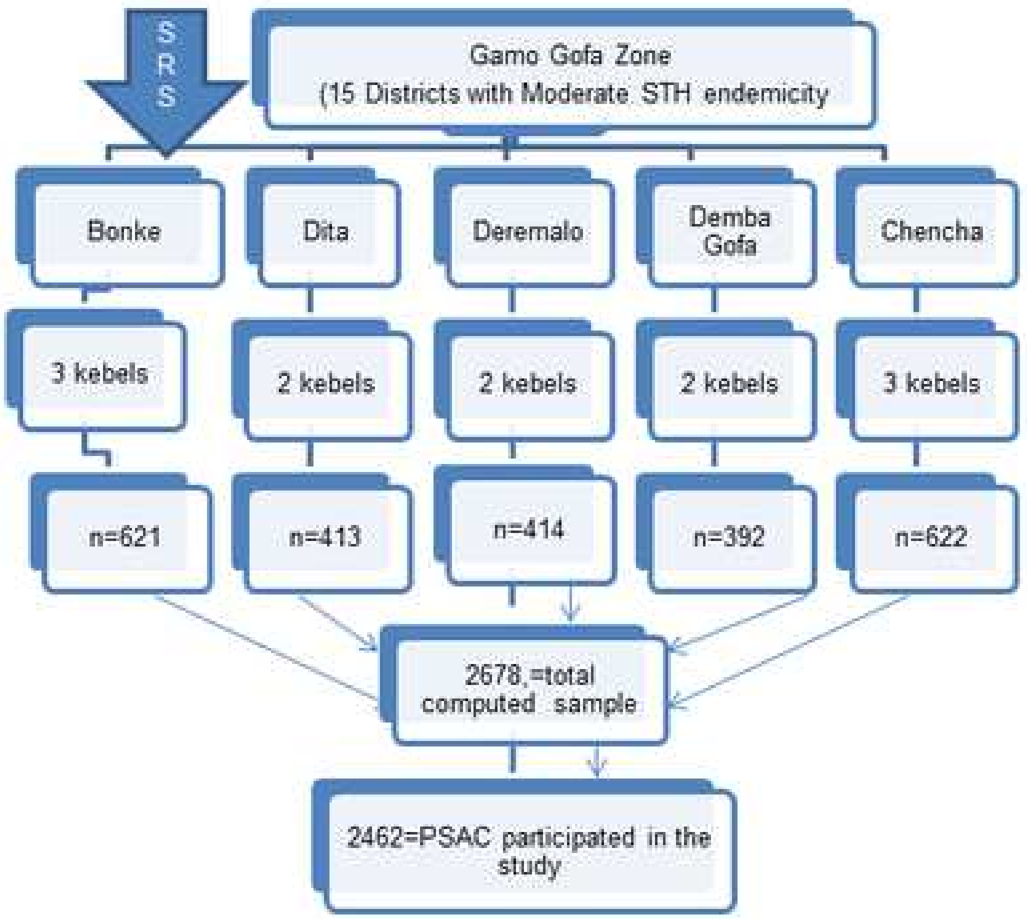

Figure

Click here to access/download;Figure;Fig 2.tif

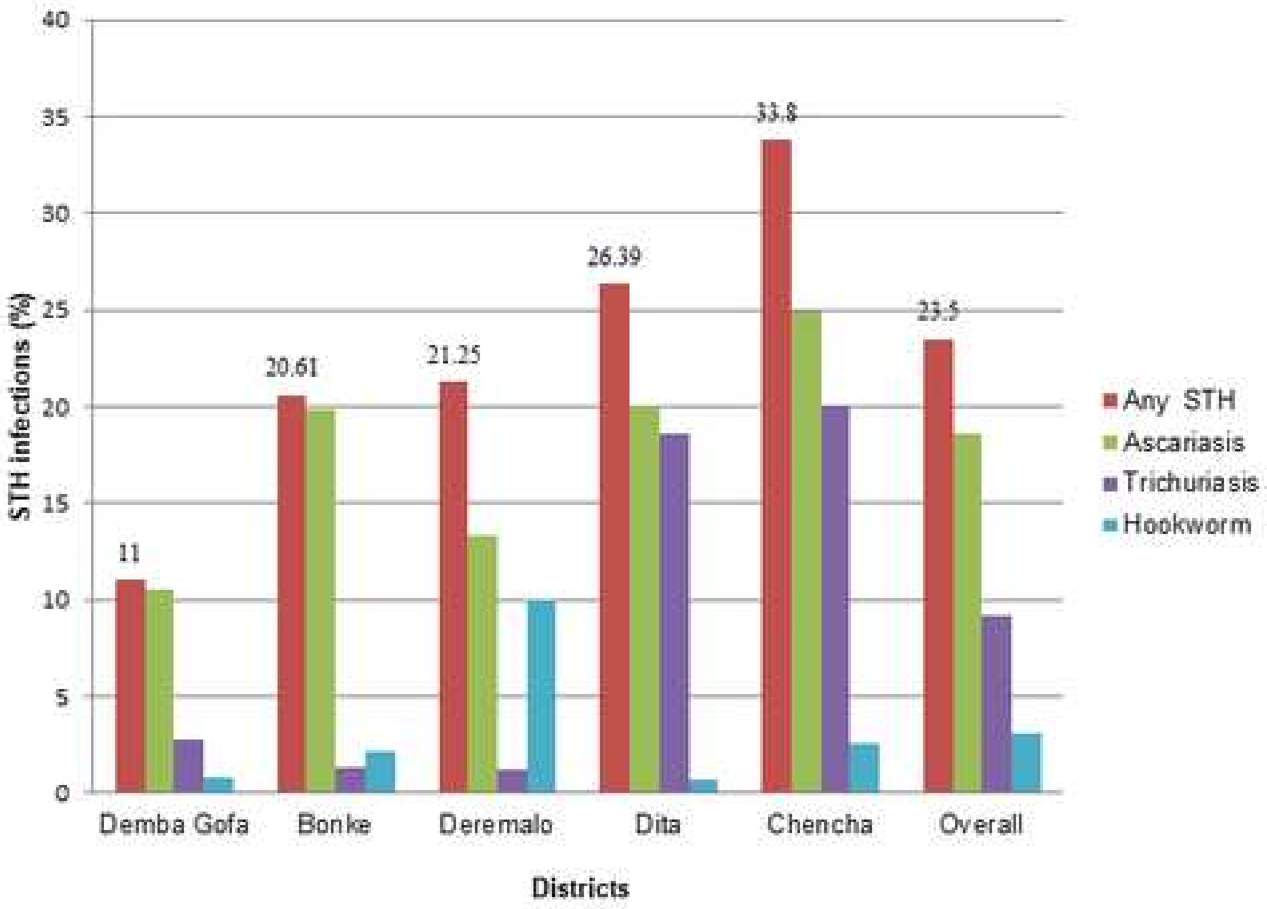

Supplement: Attachment — Submitted filename: LRLPONE-D-20-25387_reviewer.pdf [file pone.0243946.s002.pdf]
